# Squalene alleviates DSS-induced colitis by restoring Nrf2-dependent redox balance and gut microbiota homeostasis

**DOI:** 10.3389/fphar.2026.1938047

**Published:** 2026-09-18

**Authors:** Peng Jiang, Zhao Ling, Shuo Han, Yake Wang, Jing Wang

**Affiliations:** 1 College of Animal Science and Technology, Tarim University, Alaer, China; 2 State Key Laboratory for Diagnosis and Treatment of Severe Zoonotic Infectious Diseases, Institute of Zoonosis, and College of Veterinary Medicine, Jilin University, Changchun, China; 3 Engineering Laboratory of Tarim Animal Diseases Diagnosis and Control, Xinjiang Production & Construction Corps, Alaer, China; 4 Hangzhou Xiaoshan Donghai Aquaculture Co., Ltd., Hangzhou, Zhejiang Province, China

**Keywords:** gut microbiota, intestinal barrier, Nrf2, oxidative stress, squalene, ulcerative colitis

## Abstract

**Background:**

Ulcerative colitis (UC) is a chronic inflammatory disorder characterized by excessive oxidative stress, intestinal epithelial barrier dysfunction, and gut microbiota dysbiosis; despite expanding therapeutic options, achieving durable disease control remains challenging. Squalene (SQ), a naturally occurring triterpene with antioxidant and anti-inflammatory properties, has emerged as a promising bioactive compound. Although the protective potential of SQ in experimental colitis has been previously reported, the molecular dependence of this effect and the functional contribution of the gut microbiota remain incompletely defined. This study investigated the protective effects of SQ against DSS-induced experimental colitis and the roles of Nrf2 signaling and the gut microbiota in mediating these effects.

**Methods:**

DSS-induced colitis was established in mice to evaluate the protective effects of SQ. Disease severity, histopathological injury, oxidative stress, inflammatory responses, intestinal barrier integrity, and gut microbiota composition were assessed. Nrf2-deficient mice, pharmacological inhibition of Nrf2, antibiotic-mediated microbiota depletion, fecal microbiota transplantation (FMT), 16S rRNA sequencing, cellular thermal shift assay (CETSA), and lipopolysaccharide-stimulated Caco-2 cells were employed to investigate the underlying mechanisms.

**Results:**

SQ markedly alleviated experimental colitis by reducing disease activity, preventing colon shortening, and improving histopathological injury. Mechanistically, SQ activated Nrf2-dependent antioxidant defense, attenuated oxidative stress, suppressed NF-κB-mediated inflammation, and restored intestinal barrier integrity by enhancing tight junction protein expression. These protective effects were substantially diminished in Nrf2-deficient mice, identifying Nrf2 as a critical mediator of SQ activity. SQ also reshaped gut microbial composition and partially restored microbial community structure. Antibiotic-mediated microbiota depletion markedly weakened the beneficial effects of SQ, whereas fecal microbiota transplantation from SQ-treated donors transferred protection against DSS-induced colitis, supporting a functional contribution of microbiota remodeling to SQ-mediated protection. Consistently, SQ suppressed inflammatory responses and restored barrier-related protein expression in lipopolysaccharide-stimulated Caco-2 cells, effects that were attenuated by pharmacological inhibition of Nrf2. CETSA further demonstrated enhanced thermal stability of Nrf2 following SQ treatment, suggesting potential target engagement between SQ and Nrf2.

**Conclusion:**

SQ exerts protective effects against experimental colitis primarily through an Nrf2-dependent host molecular axis, with gut microbiota remodeling providing an important complementary functional contribution. These findings identify Nrf2 as the principal molecular mediator of SQ activity and position microbiota remodeling as a complementary component of its protective effects, supporting SQ as a promising bioactive candidate for intestinal inflammatory disorders.

## 1 Introduction

Ulcerative colitis (UC) is a chronic, relapsing inflammatory disorder of the colon and rectum characterized by continuous mucosal inflammation, leading to symptoms such as diarrhea, rectal bleeding, and abdominal pain (Gros and Kaplan, 2023). The global burden of UC has increased substantially over recent decades, with a particularly rapid rise in newly industrialized regions, including parts of Asia, highlighting its growing public health significance (Ng et al., 2017). Although the etiology of UC remains incompletely understood, it is widely accepted to involve a complex interplay of genetic susceptibility, environmental factors, intestinal barrier dysfunction, dysregulated immune responses, and gut microbiota imbalance (Porter et al., 2020). Among these, disruption of the epithelial barrier is considered a critical pathogenic feature that can facilitate luminal antigen translocation, thereby perpetuating intestinal inflammation. Current therapeutic strategies, including aminosalicylates, corticosteroids, immunosuppressants, biologic agents, and small-molecule therapies, can induce and maintain remission in a proportion of patients (Luo et al., 2022). Nevertheless, a substantial proportion of patients fail to achieve or maintain durable disease control despite available therapies, highlighting the continued need for safe and effective therapeutic strategies (Gros and Kaplan, 2023). Therefore, identifying pharmacologically active compounds capable of simultaneously regulating inflammation, restoring epithelial integrity, and maintaining intestinal homeostasis represents an important strategy for developing novel interventions for UC.

Oxidative stress, characterized by excessive production of reactive oxygen species (ROS) and disruption of redox homeostasis, is a critical contributor to the pathogenesis of UC (Bhattacharyya et al., 2014). Accumulating evidence indicates that oxidative stress not only induces epithelial damage but also amplifies inflammatory responses, thereby perpetuating mucosal injury. Nuclear factor erythroid 2-related factor 2 (Nrf2) is a master regulator of cellular antioxidant defense and is activated under oxidative stress to restore redox balance and protect against tissue injury (Nguyen et al., 2009). In contrast, nuclear factor-κB (NF-κB) functions as a central mediator of inflammation by promoting the transcription of pro-inflammatory cytokines (Kordjazy et al., 2018). Notably, crosstalk between Nrf2 and NF-κB pathways plays a pivotal role in regulating the balance between oxidative stress and inflammation. Therefore, targeting the Nrf2/NF-κB axis represents a promising strategy for the management of UC.

The gut microbiota constitutes a highly dynamic ecosystem that plays a fundamental role in maintaining intestinal homeostasis. Increasing evidence has implicated microbial dysbiosis as a critical factor in the pathogenesis of UC, typically characterized by reduced microbial diversity, depletion of beneficial commensals, and expansion of pro-inflammatory microorganisms (Bethlehem et al., 2024). Such alterations can perturb the production of microbiota-derived metabolites, including short-chain fatty acids, impair mucosal barrier integrity, and promote aberrant immune activation, thereby sustaining intestinal inflammation (Zhang et al., 2022). Importantly, functional studies in gnotobiotic mice have demonstrated that IBD-associated dysbiotic microbiota can promote pro-inflammatory host responses and increase susceptibility to intestinal inflammation (Nagao-Kitamoto et al., 2016; Britton et al., 2019). In parallel, microbiota-directed interventions, including FMT, have shown therapeutic potential in UC, although efficacy remains variable (Lopetuso et al., 2023). Nevertheless, these findings underscore the pivotal role of gut microbiota as both a regulator and a therapeutic target in UC. Therefore, determining whether pharmacologically active compounds exert their beneficial effects through microbiota-dependent mechanisms is critical for understanding their mechanisms of action.

Squalene (SQ) is a naturally occurring triterpene widely distributed in plants, animals, and humans and serves as an intermediate in sterol biosynthesis (Micera et al., 2020). Beyond its physiological role in cholesterol metabolism, SQ has attracted pharmacological interest due to its free radical-scavenging capacity and ability to regulate inflammatory signaling pathways. Accumulating evidence has demonstrated that SQ possesses diverse biological activities, including antioxidant, anti-inflammatory, and cytoprotective effects (Micera et al., 2020; Luke et al., 2024). Previous work has shown that dietary SQ can attenuate DSS-induced acute colitis, primarily in association with suppression of p38 MAPK/NF-κB signaling (Sanchez-Fidalgo et al., 2015). However, the molecular dependence of this protection and the functional contribution of the gut microbiota remain incompletely defined, particularly with respect to Nrf2-mediated redox regulation and host–microbiota interactions. Accordingly, it remains to be determined whether SQ-mediated protection depends on Nrf2 signaling and whether gut microbiota remodeling contributes functionally to this effect. Addressing these questions is essential for a comprehensive understanding of the therapeutic potential of SQ. In the present study, we systematically investigated the protective effects and underlying mechanisms of SQ in dextran sulfate sodium (DSS)-induced colitis using both *in vivo* and *in vitro* models. Specifically, we investigated whether SQ-mediated protection depends on Nrf2 signaling using Nrf2-deficient mice and assessed the functional contribution of microbiota remodeling through antibiotic depletion and FMT approaches. Complementary experiments in lipopolysaccharide-stimulated Caco-2 cells were performed to further evaluate the epithelial protective effects of SQ on intestinal epithelial cells.

## 2 Materials and methods

### 2.1 Chemicals and reagents

Dextran sulfate sodium (DSS; molecular weight 36,000–50,000 Da) was purchased from MP Biomedicals (OH, USA). Squalene (SQ; purity ≥98%, Cat. No. HY-N1214) was obtained from MedChemExpress (NJ, USA) (Figure 1A). The Nrf2 inhibitor ML385 (Cat. No. M10268) and 5-aminosalicylic acid (5-ASA; Cat. No. M10863) were purchased from Psaitong (Beijing, China). Lipopolysaccharide (LPS; *Escherichia coli* O55:B5) was sourced from Sigma-Aldrich (MO, USA). Primary antibodies against GAPDH, Nrf2, p65, p-p65, IκBα, and p-IκBα were obtained from Abcam (Cambridge, UK). Primary antibodies against COX-2, ZO-1, occludin, HO-1, and NQO1 were purchased from Proteintech (Wuhan, China). Detailed information on the primary antibodies, including manufacturers, catalog numbers, and working dilutions, is provided in Supplementary Table S1. Unless otherwise stated, all chemicals and reagents used in this study were of analytical grade.

**FIGURE 1:**
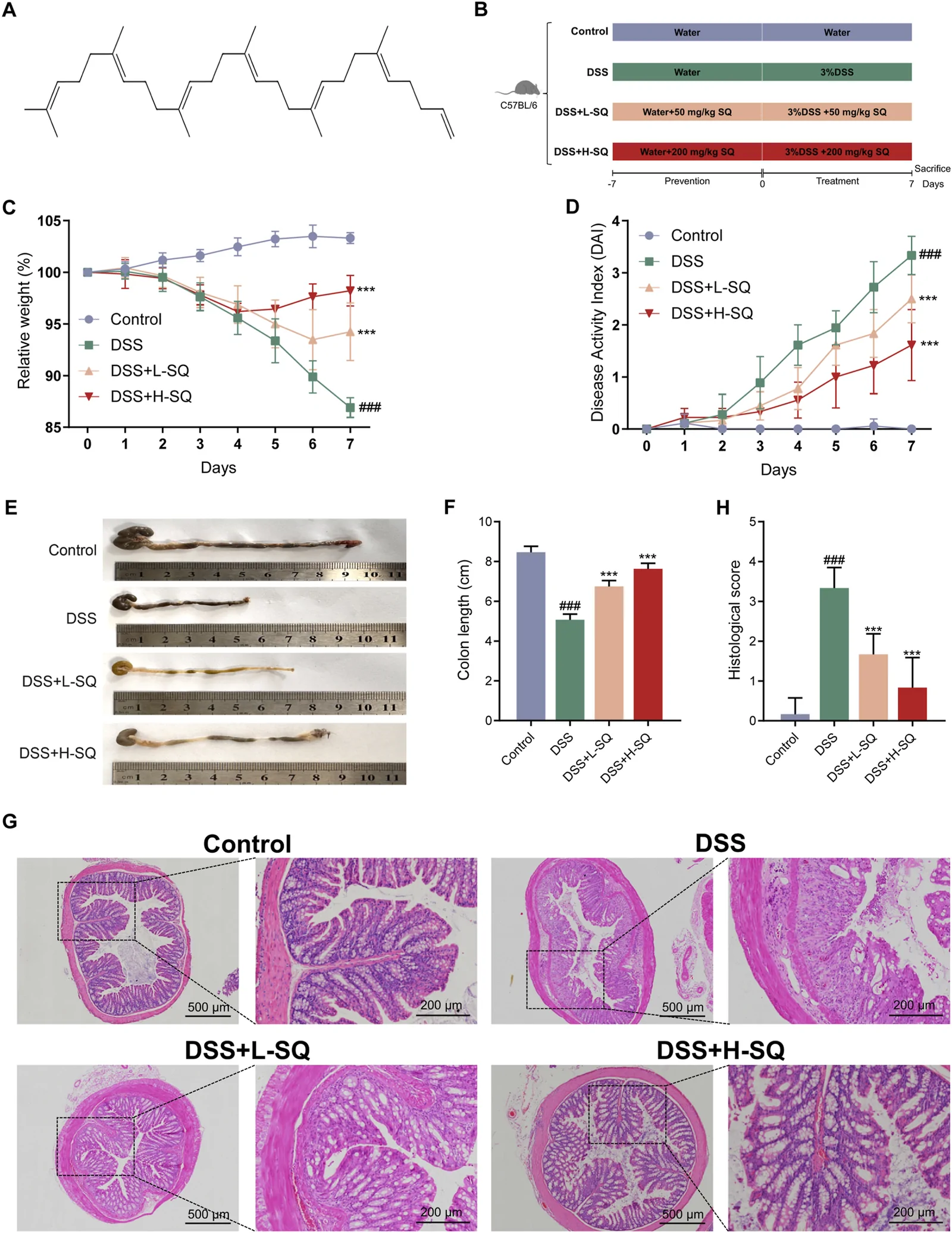
SQ alleviates DSS-induced colitis symptoms in mice. **(A)** Chemical structure of SQ. **(B)** Schematic diagram of the experimental design and treatment protocol. **(C)** Changes in relative body weight (%) during DSS administration (day 0–7). **(D)** Disease activity index (DAI) scores. **(E)** Representative images of colons from each group. **(F)** Quantitative analysis of colon length. **(G)** Representative hematoxylin and eosin (H&E) staining of colonic tissues. The right panels show enlarged views of the boxed regions in the left panels. Scale bars = 500 μm (left panels) and 200 μm (right panels). **(H)** Histopathological scoring of colonic injury. Data are presented as mean ± SD (n = 6). ^###^ *P* < 0.001 vs. Control group. ^***^ *P* < 0.001 vs. DSS group.

### 2.2 Animal experiments

An overview of all animal experiments, including group allocation, treatment schedules, and the specific purpose of each experimental cohort, is provided in Supplementary Figure S1. Five independent animal experiments were conducted: (1) evaluation of the dose-dependent protective effects of SQ in DSS-induced colitis; (2) comparison of H-SQ with the clinically used positive control 5-ASA; (3) assessment of the requirement for Nrf2 using WT and Nrf2-KO mice; (4) evaluation of the contribution of gut microbiota using antibiotic-mediated microbiota depletion; and (5) assessment of the transferability of SQ-associated microbiota-mediated protection using FMT. Detailed procedures for each experiment are described below.

#### 2.2.1 Animal experiment 1: Establishment of the UC mouse model and SQ treatment

Male C57BL/6 mice (6–8 weeks old, 19–21 g) were obtained from a certified vendor and housed under specific pathogen-free (SPF) conditions with a controlled environment (22 °C ± 2 °C, 50%–60% humidity) and a 12 h light/dark cycle. Mice had free access to standard chow and water and were allowed to acclimate for 7 days prior to experimentation. Following acclimation, mice were randomly assigned to four groups (n = 6 per group) (Figure 1B): Control, DSS, DSS + low-dose squalene (DSS + L-SQ), and DSS + high-dose squalene (DSS + H-SQ). SQ was freshly suspended in a vehicle consisting of 10% (v/v) DMSO and 90% (v/v) of a 20% (w/v) sulfobutylether-β-cyclodextrin (SBE-β-CD) solution prepared in sterile saline immediately before use. The SQ doses were selected with reference to pharmacologically active oral dose ranges previously reported in mouse studies. Oral SQ administration at 100–200 mg/kg has been reported to exert biological activity in different experimental mouse models (Sakul et al., 2019; Kim et al., 2026). Accordingly, 200 mg/kg was selected as the high dose, whereas 50 mg/kg, representing a fourfold lower exposure, was included as the low dose to provide a sufficiently separated dose range for assessing dose responsiveness. SQ was administered once daily by oral gavage at 50 or 200 mg/kg in a total volume of 200 μL per mouse. Control and DSS mice received an equal volume of the corresponding vehicle. During the pretreatment phase, mice in the L-SQ and H-SQ groups received SQ for 7 consecutive days, whereas mice in the Control and DSS groups received an equal volume of the corresponding vehicle. Subsequently, acute colitis was induced by administration of 3% (w/v) DSS in drinking water for 7 days. During DSS exposure, SQ administration was continued in the treatment groups at the same doses, while the Control group received regular drinking water throughout the experiment. Body weight, stool consistency, and the presence of fecal blood were monitored daily. Disease activity was evaluated using the disease activity index (DAI), which was calculated based on body weight loss, stool consistency, and fecal bleeding according to a previously established scoring system (Zhu et al., 2022). Detailed scoring criteria are provided in Supplementary Table S2. At the end of the experimental period, mice were deeply anesthetized with sodium pentobarbital (50 mg/kg body weight, intraperitoneal injection), and euthanasia was subsequently performed by cervical dislocation. Colon tissues were collected immediately for subsequent biochemical, histological, and molecular analyses.

#### 2.2.2 Animal experiment 2: Positive-control validation with 5-ASA

To provide a pharmacological benchmark for the protective efficacy of SQ, a separate positive-control validation experiment was conducted in male C57BL/6 mice. Following acclimation, mice were randomly assigned to four concurrently studied groups (n = 6 per group): Control, DSS, DSS + H-SQ, and DSS+5-ASA. DSS induction and H-SQ administration were performed according to the regimen described in Section 2.2.1. The DSS+5-ASA group received 5-ASA (100 mg/kg) by oral gavage once daily for 7 days before DSS exposure and throughout the subsequent 7-day DSS challenge, matching the intervention schedule used for H-SQ. The 5-ASA dose was selected with reference to previous studies using oral 5-ASA in DSS-induced experimental colitis (da Silva et al., 2016). Body weight and DAI were monitored daily, and colon length, histopathological injury, and colonic levels of IL-1β, IL-6, TNF-α, and IL-10 were evaluated at the experimental endpoint using the procedures described above.

#### 2.2.3 Animal experiment 3: DSS-induced colitis model in WT and Nrf2-KO mice with SQ administration

To further validate the involvement of Nrf2 in the effects of SQ, wild-type (WT) and Nrf2-knockout (Nrf2-KO) mice were used to establish DSS-induced colitis models. Nrf2-KO mice (C57BL/6 background) were kindly provided by Prof. Kai Zhang (The Second Hospital of Jilin University, Changchun, China). Mice of each genotype were randomly divided into two groups (n = 6 per group): DSS and DSS + H-SQ groups. The treatment regimen, including SQ pretreatment and DSS induction, was performed as described in Section 2.2.1. Briefly, mice in the DSS + H-SQ groups received SQ (200 mg/kg by oral gavage) once daily for 7 days before DSS exposure and throughout the subsequent DSS challenge, whereas the corresponding DSS groups received vehicle. All mice received 3% (w/v) DSS in the drinking water for 7 consecutive days to induce acute colitis.

#### 2.2.4 Animal experiment 4: Antibiotic-mediated microbiota depletion and SQ treatment

To evaluate the contribution of gut microbiota to the effects of SQ, an antibiotic (ABX)-mediated microbiota depletion model was established. Mice were randomly assigned to three groups (n = 6 per group) under antibiotic treatment: Control, DSS, and DSS + H-SQ groups. All mice received a broad-spectrum antibiotic cocktail in drinking water throughout the 14-day experimental period, consisting of vancomycin (0.5 g/L), metronidazole (1 g/L), neomycin sulfate (1 g/L), and ampicillin (1 g/L). During the first 7 days, SQ (200 mg/kg by oral gavage) was administered to the DSS + H-SQ group, while the remaining groups received vehicle. Subsequently, 3% (w/v) DSS was administered in the drinking water for 7 days to the DSS and DSS + H-SQ groups, with SQ administration continued throughout the DSS challenge.

#### 2.2.5 Animal experiment 5: Fecal microbiota transplantation (FMT)

To investigate the functional contribution of gut microbiota to SQ-mediated protection, fecal microbiota transplantation (FMT) was performed. Donor fecal samples were collected under sterile conditions from mice in the Control, DSS, and DSS + H-SQ groups described in Section 2.2.1. Fresh fecal samples from all donor mice within each group were pooled, and 100 mg of the pooled feces was suspended in 1 mL sterile saline, thoroughly homogenized, and centrifuged at 800 *g* for 3 min to remove large particulate matter. The fecal suspension was freshly prepared before each transplantation, and the resulting supernatant was used for gavage. Recipient mice were pretreated with the same broad-spectrum antibiotic cocktail described in Section 2.2.4 for 7 consecutive days to deplete the endogenous gut microbiota. Following antibiotic pretreatment, mice were randomly assigned to three groups (n = 6 per group): FMT-Control, FMT-DSS, and FMT-DSS + H-SQ. Recipient mice received 200 μL of the corresponding fecal suspension by oral gavage once daily for 14 consecutive days. During the final 7 days of FMT, acute colitis was induced by administration of 3% (w/v) DSS in the drinking water.

### 2.3 Histological staining and analysis

Distal colon tissues were fixed in 4% paraformaldehyde for 24 h at room temperature, followed by paraffin embedding and sectioning at 4 μm thickness. Sections were subjected to hematoxylin and eosin (H&E) staining to evaluate histopathological alterations and Alcian blue-periodic acid-Schiff (AB-PAS) staining to assess goblet cell abundance and mucin secretion, according to the manufacturer’s instructions (Solarbio, Beijing, China). Immunohistochemical staining for Muc2 was performed as previously described (Bian et al., 2024). Representative images were acquired using an Olympus VS200 digital slide scanner (Evident, Tokyo, Japan), and multiple non-overlapping random fields from each section were analyzed. Histopathological damage was evaluated in a blinded manner according to a previously established histopathological scoring system incorporating inflammatory cell infiltration, the extent of inflammation, and crypt architectural disruption (Horino et al., 2008; Jiang et al., 2024). Detailed histopathological scoring criteria are summarized in Supplementary Table S3. AB-PAS-positive goblet cells were quantified using ImageJ software (National Institutes of Health, USA). At least five well-oriented crypts from each section were analyzed.

### 2.4 Immunofluorescence analysis

Immunofluorescence staining was performed on paraffin-embedded colon sections and Caco-2 cells to evaluate ZO-1 expression. For tissue sections, antigen retrieval was conducted using citrate buffer, followed by blocking with 5% BSA. For cell staining, cells were fixed with 4% paraformaldehyde, permeabilized, and blocked under standard conditions. Samples were then incubated with primary antibody against ZO-1 overnight at 4 °C, followed by incubation with an Alexa Fluor 488-conjugated secondary antibody (Invitrogen, USA). Nuclei were counterstained with DAPI. Fluorescence images were acquired using a fluorescence microscope (Nikon, Japan). For each sample, representative fields were captured, and fluorescence intensity was semi-quantitatively analyzed using ImageJ software.

### 2.5 Cytokine determination

Colonic tissues were homogenized in cold PBS and centrifuged to obtain supernatants for cytokine analysis. The levels of the pro-inflammatory cytokines IL-1β, IL-6, and TNF-α and the anti-inflammatory cytokine IL-10 were quantified using commercial ELISA kits according to the manufacturers’ instructions. Specifically, the IL-6 ELISA kit was obtained from BD Biosciences (San Diego, CA, USA), while the kits for IL-1β, TNF-α, and IL-10 were purchased from Elabscience (Wuhan, China). Absorbance was measured at 450 nm using a microplate reader (BioTek, WA, USA).

### 2.6 Oxidative stress analysis

Colonic tissue homogenates were prepared as described above, and the supernatants were collected for biochemical analysis. The activities of antioxidant enzymes, including superoxide dismutase (SOD), catalase (CAT), and glutathione peroxidase (GSH-Px), as well as the level of malondialdehyde (MDA), were determined using commercial assay kits according to the manufacturers’ instructions. Specifically, the MDA assay kit was obtained from Nanjing Jiancheng Bioengineering Institute (Nanjing, China), while the kits for SOD, CAT, and GSH-Px were purchased from Solarbio.

### 2.7 Western blot assay

Colonic tissues and cells were lysed in ice-cold RIPA lysis buffer containing protease and phosphatase inhibitors, and the resulting lysates were incubated on ice for 30 min. Lysates were centrifuged at 12,000 × g for 10 min at 4 °C, and the supernatants were collected for protein analysis. Protein concentration was determined using a Bradford Protein Assay Kit (Beyotime, China). Equal amounts of protein were separated by SDS-PAGE and transferred onto PVDF membranes (Millipore). After blocking with 5% non-fat milk in TBST, membranes were incubated overnight at 4 °C with primary antibodies. After washing, membranes were incubated with HRP-conjugated secondary antibodies for 2 h at room temperature. Protein bands were visualized using an enhanced chemiluminescence (ECL) system and quantified using ImageJ software. GAPDH was used as the loading control.

### 2.8 Cell culture and *in vitro* assays

Human intestinal epithelial Caco-2 cells (ATCC, Manassas, VA, USA) were maintained in high-glucose Dulbecco’s Modified Eagle Medium supplemented with 10% fetal bovine serum and 1% penicillin-streptomycin at 37    °C in a humidified 5% CO_2_ atmosphere. Cell viability was determined using a Cell Counting Kit-8 (Glpbio, USA). Cells were treated with SQ (0–400 μM) for 24 h to assess cytotoxicity. For mechanistic evaluation, an inflammatory injury model was established using 1 μg/mL lipopolysaccharide (LPS) for 24 h, with or without SQ (25, 50, and 100 μM) co-treatment. The LPS concentration was selected based on previous Caco-2 studies in which 1 μg/mL LPS reliably induced inflammatory responses and epithelial barrier dysfunction while remaining suitable for intervention experiments (Wei et al., 2022; Garcia et al., 2024). In selected experiments, cells were pretreated with the Nrf2 inhibitor ML385 (10 μM) for 2 h prior to LPS and SQ exposure. Cells were subsequently harvested for analyses of inflammatory cytokine expression, oxidative stress-related parameters, and tight junction proteins. To evaluate epithelial barrier function *in vitro*, Caco-2 cells were seeded onto 24-well Transwell inserts (0.4 μm pore size, 0.33 cm^2^ growth area) and cultured for 21 days to establish differentiated monolayers (Supplementary Figure S3A). Following treatment with LPS in the presence or absence of SQ (25, 50, or 100 μM), transepithelial electrical resistance (TEER) was measured using a Millicell ERS-2 Voltohmmeter (Millipore, Bedford, MA, USA), and resistance values were normalized to the membrane surface area (Ω·cm^2^). Paracellular permeability was subsequently evaluated using FITC-dextran (4 kDa, 200 μg/mL), which was added to the apical chamber for 2 h at 37 °C. Fluorescence intensity in the basolateral chamber was determined at excitation/emission wavelengths of 485/525 nm using a fluorescence microplate reader. Unless otherwise indicated, all cell-based experiments were independently repeated three times. Technical replicates within each independent experiment were averaged to generate a single value for statistical analysis; therefore, n represents the number of independent experiments rather than the number of technical replicates.

### 2.9 Quantitative real-time PCR (qRT-PCR)

Total RNA was extracted from LPS-stimulated Caco-2 cells using the RNA Extraction Kit according to the manufacturer’s instructions (Solarbio). RNA concentration and purity were determined spectrophotometrically, and equal amounts of RNA were reverse-transcribed into cDNA. Quantitative PCR was performed using Universal Blue qPCR SYBR Green Master Mix (YEASEN, China) on the qTOWER^3^ Real-Time PCR system (Analytik, Jena, Germany). Relative mRNA expression levels of inflammatory cytokines (IL-1β, IL-6, and TNF-α) were normalized to GAPDH and calculated using the 2^−ΔΔCt^ method. All reactions were performed in triplicate. Primer sequences are listed in Supplementary Table S4.

### 2.10 Cellular thermal shift assay (CETSA)

The cellular thermal shift assay (CETSA) was performed to assess potential target engagement of SQ with Nrf2 based on ligand-induced protein stabilization (Jafari et al., 2014). Caco-2 cells were treated with SQ (100 μM) or an equal volume of DMSO for 1 h. After treatment, cells were harvested, washed with ice-cold PBS, and resuspended in PBS. The cell suspensions were aliquoted into equal volumes and subjected to thermal denaturation at a gradient of temperatures (37 °C–80 °C) for 3 min using a thermal cycler, followed by cooling at 4 °C for 3 min. Subsequently, cells were lysed, and the samples were centrifuged to separate soluble fractions from precipitated proteins. The supernatants were collected and analyzed by Western blot to determine the thermal stability of Nrf2.

### 2.11 Molecular docking and molecular dynamics simulation

To further evaluate the potential interaction between SQ and Nrf2, molecular docking and molecular dynamics (MD) simulations were performed. The full-length human Nrf2 structure (UniProt Q16236) was obtained from the AlphaFold database (Jumper et al., 2021), and the three-dimensional structure of SQ was obtained from PubChem (CID 638072). Potential binding pockets were identified using fpocket 4.2.3, followed by molecular docking with AutoDock Vina 1.2.3 (Eberhardt et al., 2021). Three candidate pockets were screened using three independent random seeds with an exhaustiveness of 64, and the representative pose from pocket 4 (seed 1101, pose 004; Vina score, −5.914 kcal/mol) was selected as the initial structure for MD simulation. The protein and ligand were parameterized using the Amber ff19SB force field and GAFF2 with RESP-derived charges, respectively. The complex was solvated with TIP3P water and neutralized in approximately 0.154 M NaCl. Following energy minimization and sequential 1-ns NVT and 1-ns NPT equilibration, a 100-ns production simulation was performed using GROMACS 2025.4 at 300 K and 1 bar with a 2-fs time step. Trajectories were corrected for periodic boundary conditions, centered, and fitted to the Nrf2 backbone before analysis. Protein root-mean-square deviation (RMSD), root-mean-square fluctuation (RMSF), radius of gyration (Rg), solvent-accessible surface area (SASA), and RMSD–Rg free-energy landscape were analyzed. Ligand RMSD was calculated after backbone alignment, and the dynamic behavior of SQ within the binding region was further evaluated using the ligand–pocket center-of-mass distance and minimum heavy-atom distance, with HIS446, ARG449, LEU450, HIS453, GLU471, and ILE474 defining the key binding region. MM-PBSA calculations were performed using gmx_MMPBSA 1.6.4 on 101 frames extracted from the final 10 ns of the trajectory, together with residue-wise energy decomposition and structural interaction analysis.

### 2.12 Gut microbiota analysis by 16S rRNA sequencing

Fecal samples were collected under sterile conditions and stored at −80 °C until analysis. Microbial genomic DNA was extracted and used as a template for amplification of the V3–V4 hypervariable regions of the bacterial 16S rRNA gene using universal primers (338F/806R). Sequencing libraries were constructed and subjected to paired-end sequencing on an Illumina platform by Paisennuo Biotechnology Co., Ltd (Shanghai, China). Raw sequencing reads were processed using QIIME 2 version 2024.5. Sequence quality filtering, denoising, paired-end merging, and chimera removal were performed using DADA2, generating amplicon sequence variants (ASVs) for downstream analyses (Callahan et al., 2016). No additional sequence-similarity-based clustering of ASVs into operational taxonomic units (OTUs) was performed, and the ASV abundance table was used for subsequent analyses. Taxonomic classification was performed against the Greengenes2 database (release 2022.10). Microbial alpha and beta diversity were subsequently evaluated, and differences in community composition were assessed using ordination analyses. Discriminative bacterial taxa among groups were identified using linear discriminant analysis effect size (LEfSe).

### 2.13 Statistical analysis

All quantitative data are presented as the mean ± standard deviation (SD), unless otherwise specified. Statistical analyses were performed using GraphPad Prism version 8.0 (GraphPad Software, San Diego, CA, USA). The normality of data distribution was assessed using the Shapiro–Wilk test, and homogeneity of variance was evaluated using the Brown–Forsythe test. For comparisons involving three or more independent groups with normally distributed data and homogeneous variances, one-way analysis of variance (ANOVA) followed by Tukey’s multiple comparisons test was performed. For experiments involving two independent factors, including genotype and SQ treatment in the Nrf2-deficient mouse experiment, two-way ANOVA followed by Sidak’s multiple comparisons test was used. Changes in body weight and disease activity index (DAI) over time were analyzed using two-way repeated-measures ANOVA, with treatment and time as the two factors, followed by Sidak’s multiple comparisons test where appropriate. For data that did not satisfy the assumptions of normality or homogeneity of variance, nonparametric analysis was performed using the Kruskal–Wallis test followed by Dunn’s multiple comparisons test. For all analyses, a two-sided *P* value <0.05 was considered statistically significant. The exact sample size (n) for each experiment is specified in the corresponding figure legend.

## 3 Results

### 3.1 SQ alleviates DSS-induced colitis in mice

To evaluate the protective effect of SQ on colitis, a DSS-induced acute colitis model was established. Compared with the control group, DSS administration led to a progressive loss of body weight starting from day 3, with body weight decreasing to below 90% of the initial level by day 7 (Figure 1C). Notably, both low- and high-dose SQ treatments significantly attenuated DSS-induced weight loss, with a more pronounced effect observed in the high-dose group. Consistently, DSS exposure resulted in a marked increase in disease activity index (DAI), reflecting aggravated clinical symptoms. SQ treatment effectively mitigated this increase, as evidenced by significantly lower DAI scores in both treatment groups throughout the experimental period (Figure 1D). Colon shortening, a hallmark of DSS-induced colonic inflammation, was evident in the DSS group. In contrast, SQ administration significantly preserved colon length, as shown by both representative images and quantitative analysis (Figures 1E,F), indicating an overall attenuation of colonic injury. Histopathological examination further supported these findings. H&E staining revealed that colonic tissues from control mice displayed intact epithelial architecture, well-organized crypts, and minimal inflammatory infiltration. In contrast, DSS-treated mice exhibited severe mucosal damage, including epithelial disruption, crypt destruction, and extensive infiltration of inflammatory cells. Importantly, SQ treatment markedly alleviated these pathological alterations, as evidenced by improved mucosal integrity and reduced inflammatory infiltration (Figure 1G). Consistently, histological scoring demonstrated a significant reduction in tissue injury severity in SQ-treated groups compared with the DSS group (Figure 1H). Collectively, these results demonstrate that SQ effectively alleviates DSS-induced colitis, as reflected by improvements in clinical symptoms, gross colonic features, and histopathological features. To further benchmark the protective efficacy of H-SQ against an established pharmacological intervention, an independent validation cohort was compared with 5-ASA treatment (Supplementary Figure S2). DSS administration resulted in progressive body weight loss, increased DAI scores, colon shortening, severe histopathological injury, and marked disruption of colonic inflammatory cytokine profiles. Both H-SQ and 5-ASA significantly attenuated these DSS-induced alterations, as evidenced by improved body weight and DAI trajectories, preservation of colon length, reduced histological injury, decreased IL-1β, IL-6, and TNF-α levels, and restoration of IL-10. Direct comparison between H-SQ and 5-ASA revealed no significant differences in several major disease indices, including final body weight, DAI, histological score, and TNF-α levels, although endpoint-specific differences were observed for colon length and selected cytokines. Collectively, these findings demonstrate that H-SQ produced a robust protective phenotype against DSS-induced colitis when evaluated alongside the 5-ASA positive control.

### 3.2 SQ inhibits NF-κB pathway activation and restores inflammatory cytokine balance in DSS-induced colitis

To elucidate the anti-inflammatory mechanism of SQ, the activation status of the NF-κB signaling pathway was examined in colonic tissues. As shown in Figures 2A–D, DSS exposure markedly activated this pathway, as evidenced by increased phosphorylation levels of p65 and IκBα, along with elevated expression of the downstream effector COX-2 compared with the control group. Notably, SQ treatment effectively suppressed DSS-induced NF-κB activation. Both low- and high-dose SQ reduced the phosphorylation of p65 and IκBα and downregulated COX-2 expression, with generally greater effects observed in the high-dose group. Consistent with these protein-level changes, ELISA results revealed that DSS stimulation significantly elevated pro-inflammatory cytokines, including IL-1β, IL-6, and TNF-α, while markedly decreasing the anti-inflammatory cytokine IL-10 in colonic tissues (Figures 2E–H). SQ administration significantly reversed these alterations, as reflected by reduced levels of IL-1β, IL-6, and TNF-α, alongside restoration of IL-10 levels. Collectively, these findings indicate that SQ alleviates colonic inflammation by inhibiting NF-κB pathway activation and rebalancing pro- and anti-inflammatory cytokine production.

**FIGURE 2:**
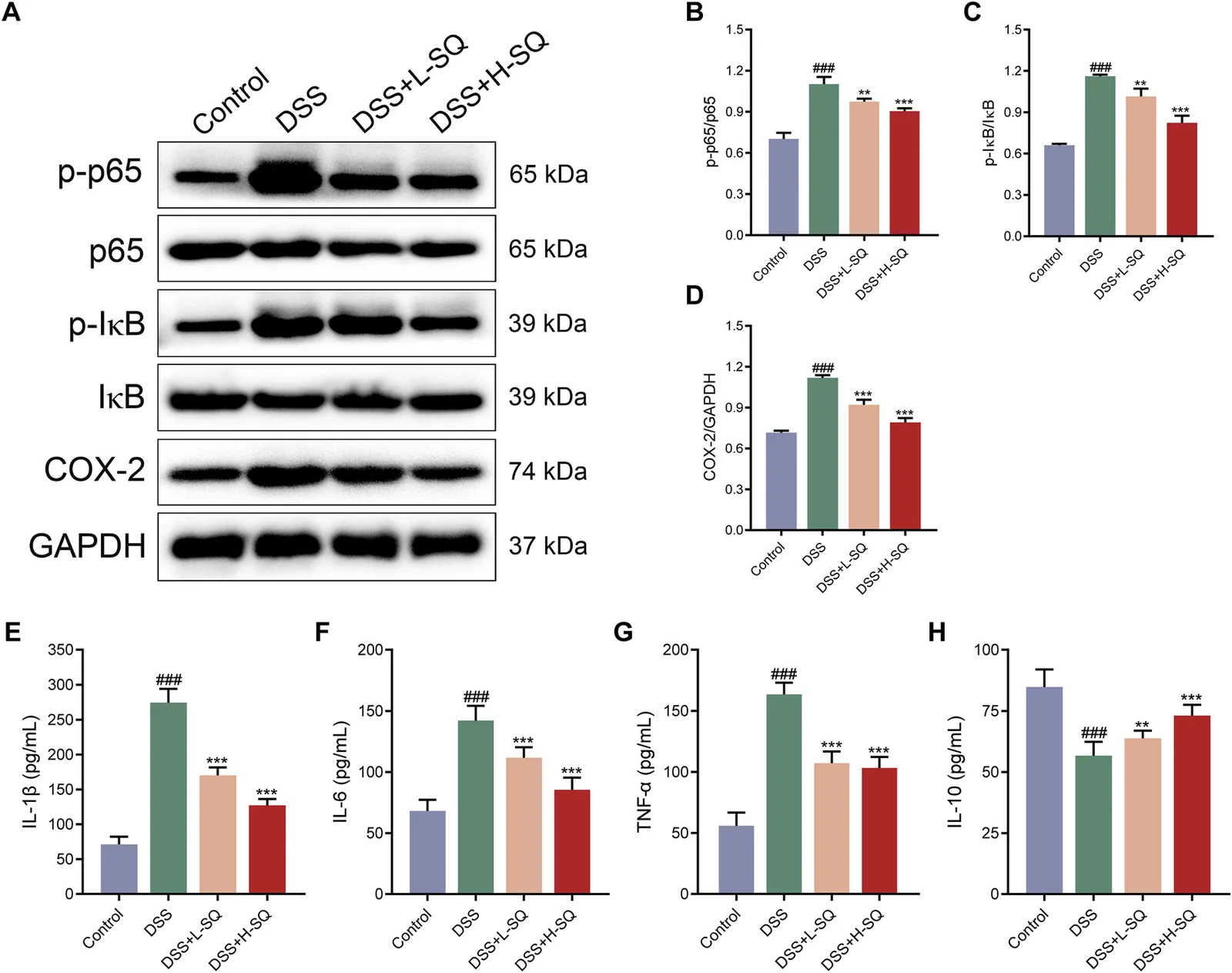
SQ suppresses NF-κB activation and modulates inflammatory cytokine production in DSS-induced colitis. **(A)** Representative Western blot images of p-p65, p65, p-IκBα, IκBα, COX-2, and GAPDH in colonic tissues. **(B–D)** Densitometric analysis of p-p65/p65 **(B)**, p-IκBα/IκBα **(C)**, and COX-2/GAPDH **(D). (E–H)** Levels of inflammatory cytokines in colonic tissues, including IL-1β **(E)**, IL-6 **(F)**, TNF-α **(G)**, and IL-10 **(H)**, determined by ELISA. Data are presented as mean ± SD (n = 6). ^###^ *P* < 0.001 vs. Control group. ^**^ *P* < 0.01, ^***^ *P* < 0.001 vs. DSS group.

### 3.3 SQ preserves intestinal barrier integrity in DSS-induced colitis

Intestinal barrier disruption is a prominent feature of ulcerative colitis and plays a pivotal role in amplifying mucosal inflammation. To determine whether SQ modulates epithelial barrier integrity, we evaluated tight junction (TJ) proteins, mucus secretion, and goblet cell abundance in colonic tissues. Western blot analysis demonstrated that DSS exposure markedly impaired epithelial junctional integrity, as evidenced by significantly reduced protein expression of ZO-1 and occludin compared with the control group (Figures 3A–C). In contrast, SQ administration restored the expression of these TJ proteins. Consistently, immunofluorescence staining further confirmed these findings at the tissue level. DSS treatment resulted in a substantial loss and discontinuous distribution of ZO-1 along the epithelial lining, reflecting compromised tight junction organization (Figures 3D,E). SQ treatment markedly enhanced ZO-1 fluorescence intensity and improved its localization pattern, suggesting restoration of epithelial barrier continuity. Given the critical role of the mucus layer as a chemical barrier, we next assessed mucin production. Immunohistochemical analysis revealed that Muc2 expression was significantly diminished in DSS-treated mice, indicating disruption of the mucus barrier (Figures 3F,G). Notably, SQ supplementation effectively increased Muc2 expression, suggesting restoration of the mucus barrier. These observations were further supported by AB-PAS staining. DSS exposure led to a pronounced depletion of goblet cells and a marked reduction in mucin-containing cells within colonic crypts (Figures 3H,I). In contrast, SQ-treated groups exhibited a significant increase in AB-PAS-positive goblet cells. These results indicate that DSS-induced colitis is associated with severe impairment of both the epithelial tight-junction barrier and mucus barrier, whereas SQ intervention effectively preserves epithelial barrier integrity by restoring tight junction protein expression and promoting mucin production.

**FIGURE 3:**
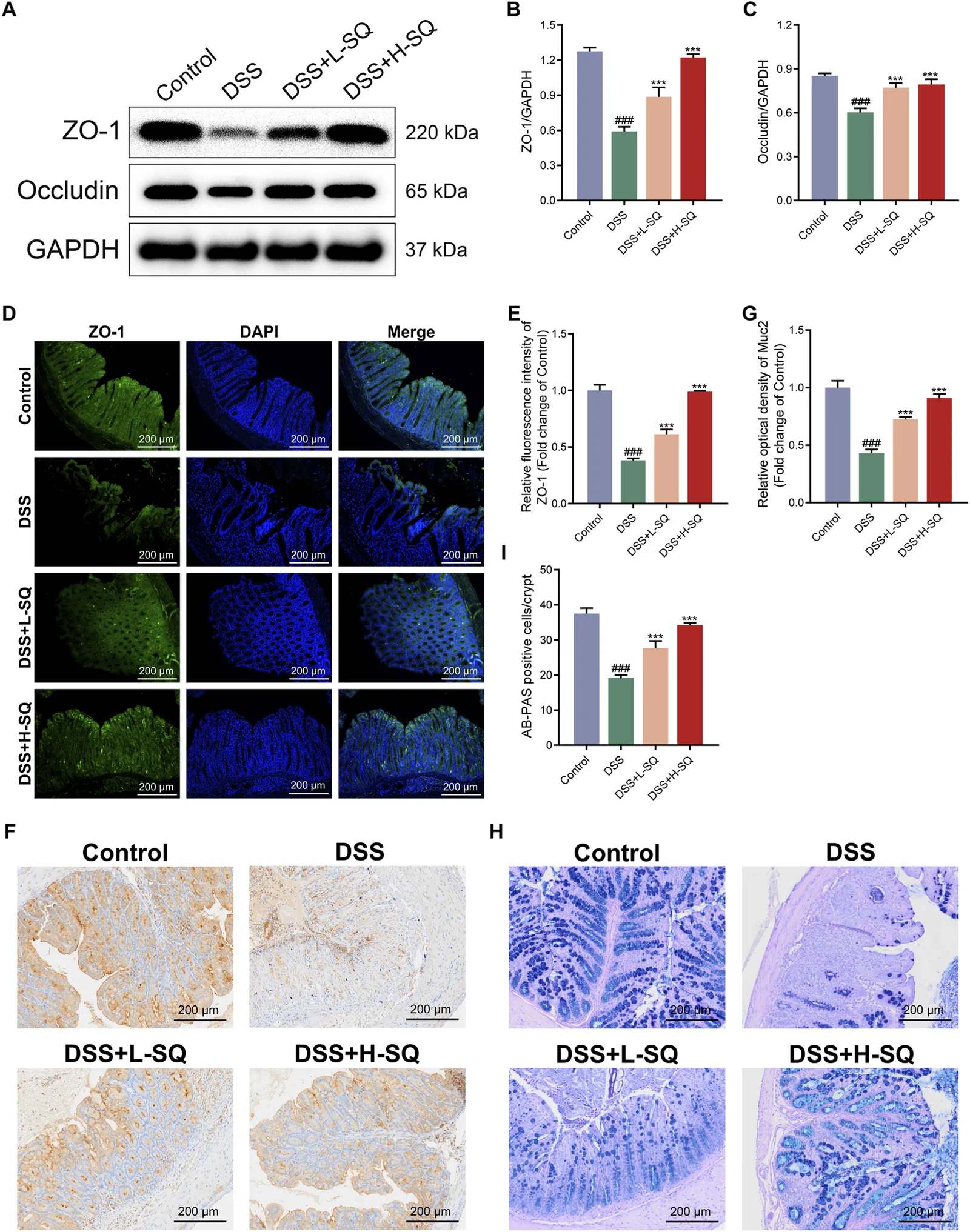
SQ preserves intestinal barrier integrity in DSS-induced colitis mice. **(A)** Representative Western blot images of tight junction proteins ZO-1 and occludin in colonic tissues. **(B,C)** Densitometric analysis of ZO-1/GAPDH **(B)** and occludin/GAPDH **(C)**. **(D)** Immunofluorescence staining of ZO-1 in colonic tissues. **(E)** Quantitative analysis of relative fluorescence intensity of ZO-1. **(F)** Immunohistochemical staining of Muc2 in colonic tissues. **(G)** Quantitative analysis of relative optical density of Muc2. **(H)** Representative Alcian blue-periodic acid-Schiff (AB-PAS) staining of colonic sections **(I)** Quantification of AB-PAS-positive goblet cells per crypt. Scale bars in **(D,F,H)** = 200 μm. Data are presented as mean ± SD (n = 6). ^###^ *P* < 0.001 vs. Control group. ^*^ *P* < 0.05, ^**^ *P* < 0.01, ^***^ *P* < 0.001 vs. DSS group.

### 3.4 SQ attenuates oxidative stress in DSS-induced colitis by activating the Nrf2 pathway

Oxidative stress is a critical contributor to the pathogenesis of ulcerative colitis, and the Nrf2 signaling pathway serves as a central regulator of cellular antioxidant defense. To investigate whether SQ modulates oxidative stress through Nrf2 activation, we first examined the expression of key proteins involved in this pathway. Western blot analysis showed that DSS exposure markedly suppressed the protein expression of Nrf2 and its downstream antioxidant enzymes, including HO-1 and NQO1, compared with the control group (Figures 4A–D). In contrast, SQ administration significantly upregulated the expression levels of Nrf2, HO-1, and NQO1, indicating activation of the Nrf2-mediated antioxidant response. To further evaluate the impact of SQ on oxidative stress status in colonic tissues, we assessed representative oxidative stress markers and antioxidant enzyme activities. As shown in Figures 4E–H, DSS treatment led to a significant increase in MDA content, accompanied by marked reductions in the activities of key antioxidant enzymes, including SOD, CAT, and GSH-Px, compared with the control group. Notably, SQ intervention effectively reversed these alterations, as evidenced by decreased MDA content and restored activities of SOD, CAT, and GSH-Px. Collectively, these findings demonstrate that DSS-induced colitis is associated with impaired antioxidant defense and excessive oxidative stress, whereas SQ treatment alleviates oxidative damage, in association with activation of the Nrf2 signaling pathway and enhancement of endogenous antioxidant capacity.

**FIGURE 4:**
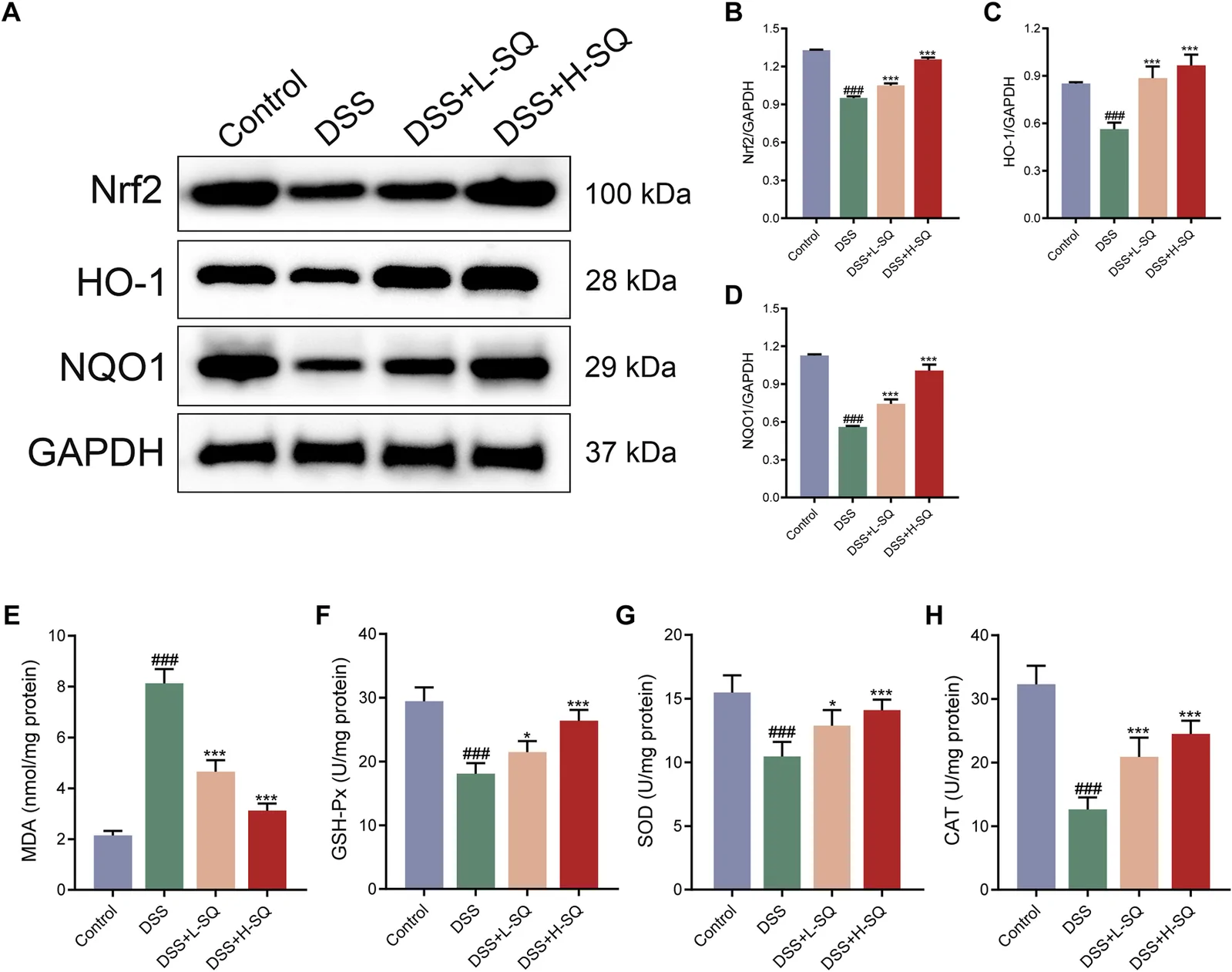
SQ activates the Nrf2 signaling pathway and alleviates oxidative stress in DSS-induced colitis. **(A)** Representative Western blot images of Nrf2 and its downstream antioxidant proteins (HO-1 and NQO1) in colonic tissues from each group. GAPDH was used as a loading control. **(B–D)** Densitometric analysis of Nrf2/GAPDH, HO-1/GAPDH, and NQO1/GAPDH protein expression levels, respectively. **(E–H)** Oxidative stress-related parameters in colonic tissues, including MDA content **(E)**, GSH-Px activity **(F)**, SOD activity **(G)**, and CAT activity **(H)**. Data are presented as mean ± SD (n = 6). ^###^ *P* < 0.001 vs. Control group. ^*^ *P* < 0.05, ^***^ *P* < 0.001 vs. DSS group.

### 3.5 Nrf2 deficiency markedly attenuates the protective effects of SQ against DSS-induced colitis

Given the central role of the Nrf2 signaling pathway in regulating oxidative stress and inflammation, we further investigated whether Nrf2 is required for the protective effects of SQ in DSS-induced colitis using Nrf2-KO mice (Figure 5A). Consistent with previous observations, DSS administration induced pronounced colitis phenotypes in WT mice, as evidenced by progressive body weight loss, elevated DAI scores, and marked colon shortening. Notably, high-dose SQ (H-SQ) treatment significantly alleviated these pathological changes in WT mice, as reflected by improved body weight trajectories, reduced DAI scores, and restoration of colon length (Figures 5B–E). Histopathological analysis further confirmed that SQ markedly attenuated DSS-induced mucosal damage, including epithelial disruption, crypt loss, and inflammatory cell infiltration, leading to a significant reduction in histological scores (Figures 5F,G). In contrast, H-SQ treatment failed to confer significant protection in Nrf2-KO mice, as no significant improvements were observed in body weight, DAI scores, or colon length relative to the DSS-treated Nrf2-KO group (Figures 5B–E). Consistently, histological examination revealed that SQ did not ameliorate mucosal injury or inflammatory infiltration in Nrf2-deficient mice, with histopathological scores remaining comparable to those of the DSS-treated Nrf2-KO group (Figures 5F,G). We next evaluated inflammatory cytokine profiles in colonic tissues. In WT mice, SQ treatment significantly suppressed DSS-induced elevations in pro-inflammatory cytokines (IL-1β, IL-6, and TNF-α) and restored the reduced level of the anti-inflammatory cytokine IL-10 (Figures 5H–K). However, these modulatory effects were markedly attenuated in Nrf2-KO mice, in which SQ administration did not significantly alter cytokine levels compared with the corresponding DSS-treated group. Similarly, assessment of oxidative stress markers revealed that SQ markedly reduced MDA accumulation and restored the activities of antioxidant enzymes, including GSH-Px, SOD, and CAT, in DSS-challenged WT mice (Figures 5L–O). In contrast, these SQ-associated improvements were not observed in Nrf2-KO mice, with no significant differences detected between the DSS and DSS + H-SQ groups. Consistent with these findings, Western blot analysis showed that H-SQ increased the expression of Nrf2 and its downstream antioxidant proteins HO-1 and NQO1 in WT mice, whereas these effects were not observed in Nrf2-KO mice (Figures 5P–S). Collectively, these results demonstrate that the protective effects of SQ against DSS-induced colitis, including its anti-inflammatory and antioxidant actions, are strongly dependent on intact Nrf2 signaling.

**FIGURE 5:**
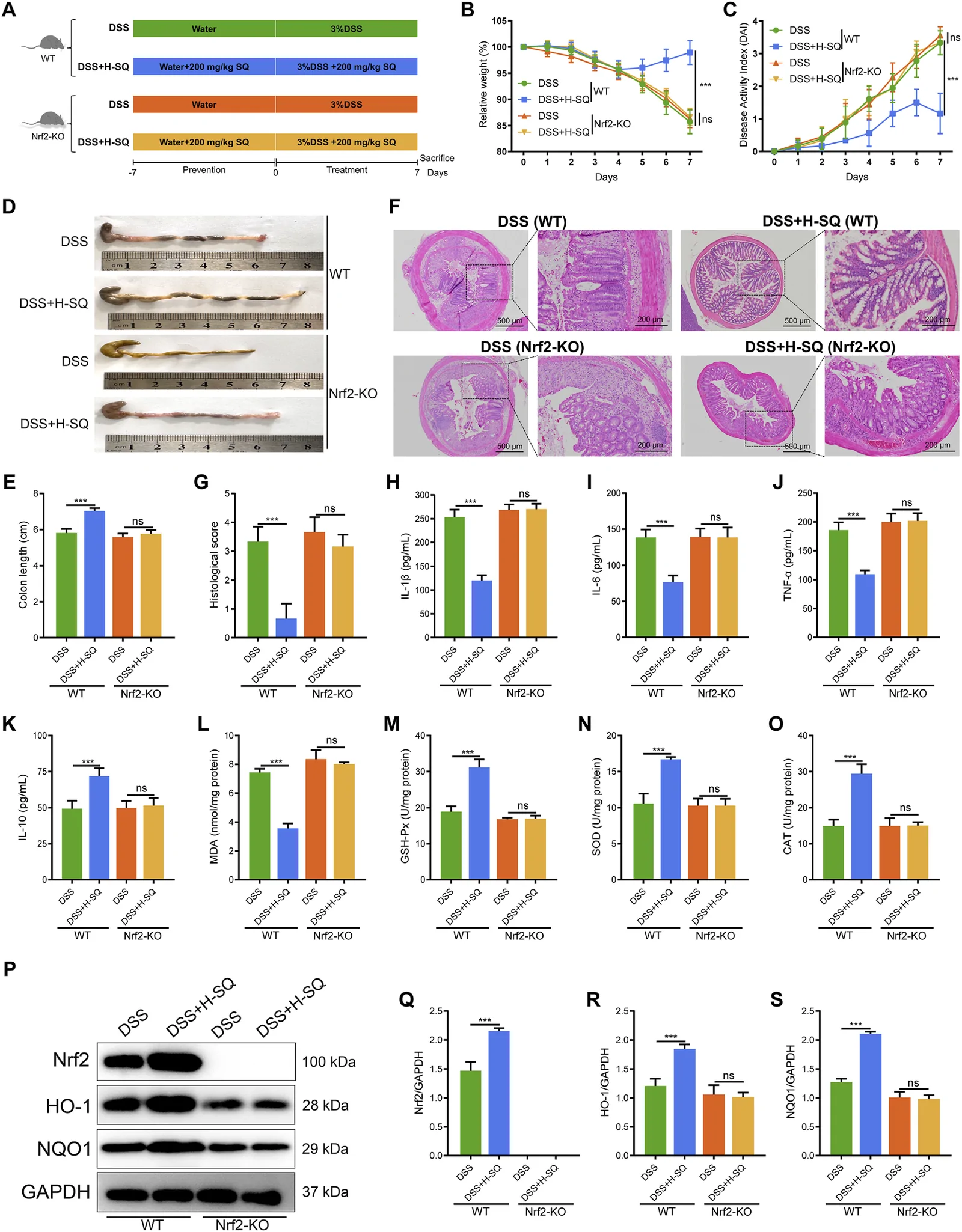
Nrf2 deficiency markedly attenuates the protective effects of SQ in DSS-induced colitis. **(A)** Schematic diagram of the experimental design for wild-type (WT) and Nrf2-KO mice subjected to DSS treatment with or without high-dose SQ (H-SQ). **(B)** Changes in body weight. **(C)** Disease activity index (DAI) scores. **(D)** Representative images of colons from each group. **(E)** Quantitative analysis of colon length. **(F)** Representative hematoxylin and eosin (H&E)-stained colon sections. Scale bars = 500 μm (left panels) and 200 μm (right panels). **(G)** Histopathological scores of colon tissues. **(H–K)** Levels of inflammatory cytokines in colon tissues, including IL-1β **(H)**, IL-6 **(I)**, TNF-α **(J)**, and IL-10 **(K)**. **(L–O)** Oxidative stress-related indicators in colon tissues, **(P)** Representative Western blot images of Nrf2 and its downstream antioxidant proteins HO-1 and NQO1 in colon tissues, with GAPDH used as a loading control. **(Q–S)** Densitometric analysis of Nrf2/GAPDH **(Q)**, HO-1/GAPDH **(R)**, and NQO1/GAPDH **(S)**, including MDA content **(L)**, GSH-Px activity **(M)**, SOD activity **(N)**, and CAT activity **(O)**. Data are presented as mean ± SD (n = 6). ^***^ *P* < 0.001. ns, not significant.

### 3.6 SQ protects against LPS-induced injury in Caco-2 cells by suppressing inflammation and preserving barrier integrity

To determine a suitable concentration range for *in vitro* experiments, the cytotoxicity of SQ in Caco-2 cells was first evaluated using the CCK-8 assay. As shown in Figure 6A, SQ exhibited no significant effect on cell viability at concentrations up to 100 μM, whereas higher concentrations (≥200 μM) induced a marked reduction in cell viability, indicating potential cytotoxicity. Based on these results, 25, 50, and 100 μM were selected as low, medium, and high concentrations, respectively, for subsequent experiments. We next assessed the protective effects of SQ on LPS-induced epithelial injury. As expected, LPS stimulation significantly decreased Caco-2 cell viability compared with the control group (Figure 6B). However, treatment with SQ at all tested concentrations markedly restored cell viability in LPS-treated cells, indicating a protective effect against LPS-induced cytotoxicity. To further evaluate the anti-inflammatory activity of SQ, the mRNA expression levels of key pro-inflammatory cytokines were measured. LPS stimulation led to a pronounced upregulation of IL-1β, IL-6, and TNF-α expression in Caco-2 cells (Figures 6C–E). In contrast, SQ treatment significantly suppressed the expression of these cytokines, with generally greater effects observed at higher SQ concentrations, demonstrating its anti-inflammatory activity *in vitro*. To further determine whether SQ functionally preserves epithelial barrier integrity, transepithelial electrical resistance (TEER) and FITC-dextran permeability assays were performed using differentiated Caco-2 monolayers. As shown in Supplementary Figure S3B,C, LPS stimulation markedly decreased TEER values while significantly increasing FITC-dextran permeability compared with the control group, indicating impaired epithelial barrier function. In contrast, SQ treatment restored TEER and reduced FITC-dextran permeability, with generally greater improvements at higher concentrations, demonstrating that SQ effectively attenuated LPS-induced barrier dysfunction. To further investigate the molecular basis underlying the improvement in barrier function, the expression of the tight junction proteins ZO-1 and occludin was examined by Western blotting. As shown in Figures 6F–H, LPS exposure markedly reduced the protein levels of ZO-1 and occludin compared with the control group, indicating disruption of epithelial barrier integrity. Notably, SQ treatment effectively restored the expression of these tight junction proteins. Consistent with the Western blot results, immunofluorescence staining further demonstrated that LPS stimulation markedly disrupted the continuous junctional distribution of ZO-1 in Caco-2 cells, as evidenced by weakened and discontinuous fluorescence signals along the cell borders (Figures 6I,J). In contrast, SQ treatment restored ZO-1 localization and fluorescence intensity, with greater recovery observed at higher SQ concentrations, resulting in a more intact and continuous junctional architecture compared with the LPS group. Collectively, these findings demonstrate that SQ protects Caco-2 cells against LPS-induced epithelial injury by suppressing inflammatory responses and preserving epithelial barrier function and integrity.

**FIGURE 6:**
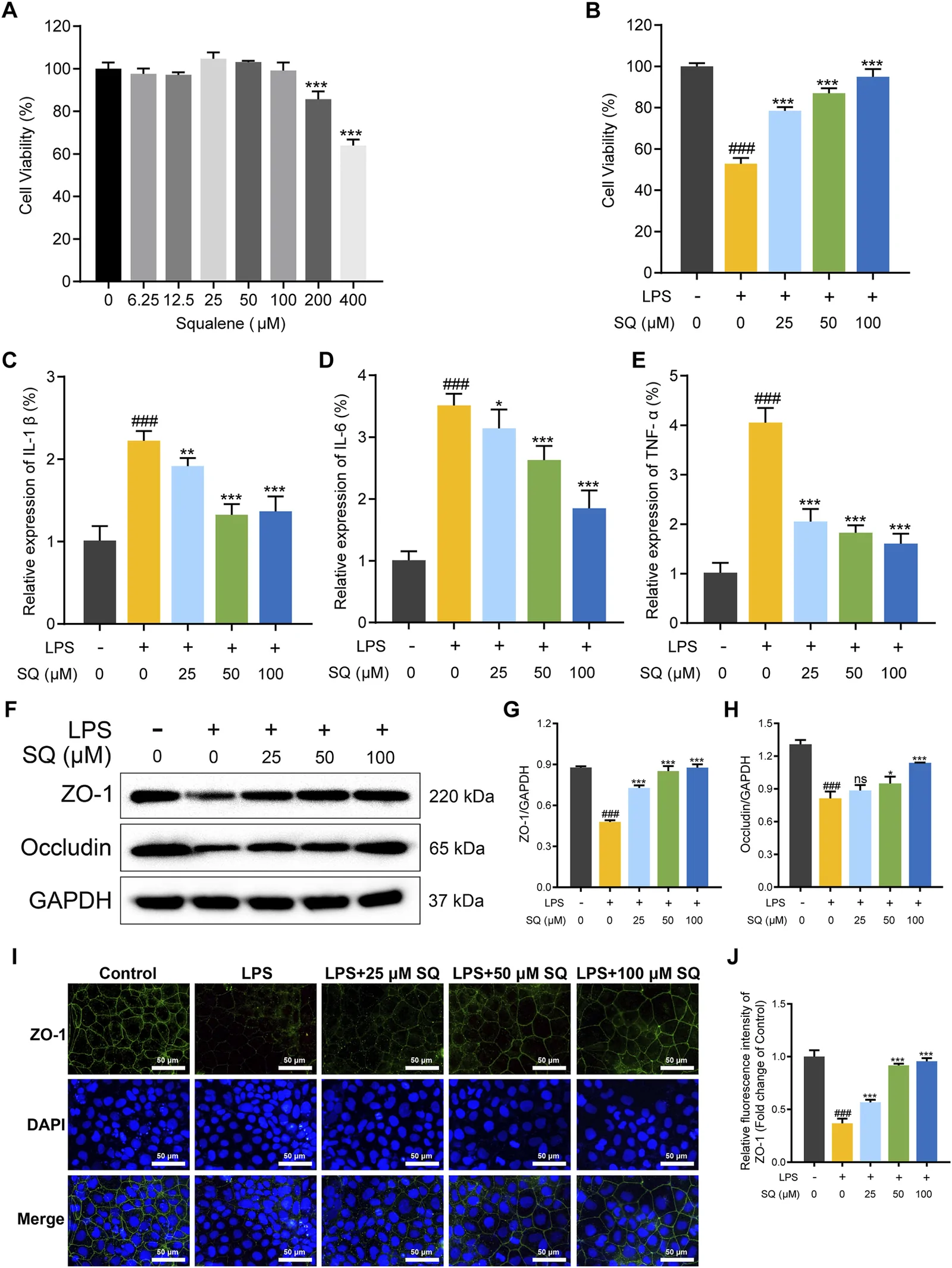
Protective effects of SQ on LPS-induced injury in Caco-2 cells. **(A)** Effects of SQ (0–400 μM) on Caco-2 cell viability assessed by CCK-8 assay. **(B)** Effects of SQ (25, 50, and 100 μM) on cell viability in LPS-stimulated Caco-2 cells. **(C–E)** Relative mRNA expression levels of pro-inflammatory cytokines IL-1β **(C)**, IL-6 **(D)**, and TNF-α **(E)** in Caco-2 cells under indicated treatments. **(F)** Western blot analysis of tight junction proteins ZO-1 and occludin in Caco-2 cells. **(G,H)** Densitometric analysis of ZO-1/GAPDH **(G)** and occludin/GAPDH **(H)**. **(I)** Immunofluorescence staining of ZO-1 in Caco-2 cells under different treatments. Scale bar = 50 μm. **(J)** Relative fluorescence intensity of ZO-1 in Caco-2 cells. Nuclei were counterstained with DAPI. Data are presented as the mean ± SD from three independent experiments (n = 3); technical replicates within each experiment were averaged before statistical analysis. ^###^ *P* < 0.001 vs. Control group. ^*^ *P* < 0.05, ^**^ *P* < 0.01, ^***^ *P* < 0.001 vs. LPS group. ns, not significant.

### 3.7 SQ exerts Nrf2-dependent antioxidant and cytoprotective effects and shows a potential interaction with Nrf2

To determine whether SQ-mediated protection involves Nrf2 signaling, LPS-stimulated Caco-2 cells were treated with the Nrf2 inhibitor ML385. As shown in Supplementary Figure S4A-D, LPS reduced the expression of Nrf2 and its downstream targets HO-1 and NQO1, and ML385 further reduced the expression of these proteins. SQ restored the expression of these proteins, whereas ML385 markedly attenuated this effect, supporting a pharmacological dependence on Nrf2 signaling. CETSA analysis further showed that SQ enhanced the thermal stability of Nrf2, with detectable protein levels maintained at higher temperatures compared with DMSO-treated cells (Supplementary Figure S4E,F), supporting potential target engagement of SQ with Nrf2. To obtain complementary structural and dynamic evidence, molecular docking followed by a 100-ns molecular dynamics (MD) simulation was performed. Docking identified pocket 4 as the preferred candidate binding region, with the representative pose showing a Vina score of −5.914 kcal/mol. Structural analysis of the MD trajectory revealed a remodeled binding region surrounding SQ, with HIS446, ARG449, LEU450, HIS453, GLU471, and ILE474 among the prominent contact residues (Supplementary Figure S5A–C). MM-PBSA analysis yielded an estimated binding energy of −26.94 ± 3.87 kcal/mol, with van der Waals interactions providing the major favorable contribution; residue-wise decomposition further identified LEU450 as a prominent favorable energetic contributor (Supplementary Figure S5D,E). During the 100-ns simulation, the full-length Nrf2 model underwent substantial early conformational relaxation before entering a relatively narrower conformational regime. Although SQ rearranged from its initial docking pose, it remained associated with the remodeled binding region during the latter half of the simulation, with mean ligand–pocket center-of-mass and minimum heavy-atom distances of 4.67 and 3.41 Å, respectively, during 50–100 ns (Supplementary Figure S6A-D). Changes in Rg and SASA, together with RMSF and RMSD–Rg free-energy landscape analyses, further characterized the conformational evolution and flexibility of the complex (Supplementary Figure S6E-H). Collectively, these computational findings provide complementary structural and dynamic support for a potential SQ–Nrf2 interaction. Consistently, functional assays demonstrated that LPS exposure significantly decreased SOD activity and cell viability while increasing MDA levels (Supplementary Figure S4G-I). SQ reversed these alterations, whereas ML385 pretreatment attenuated its protective effects, as evidenced by reduced antioxidant capacity and impaired cell viability compared with SQ treatment alone.

### 3.8 SQ reshapes gut microbial diversity and community structure in DSS-induced colitis

To determine whether the protective effects of SQ were associated with modulation of the gut microbiota, 16S rRNA gene sequencing was performed using fecal samples collected from the Control, DSS, and DSS + H-SQ groups. Venn diagram analysis revealed both shared and group-specific amplicon sequence variants (ASVs) among the three groups (Figure 7A). A total of 422 ASVs were shared among all groups, whereas the Control, DSS, and SQ groups contained 3041, 2519, and 2633 group-specific ASVs, respectively. The SQ group shared more ASVs with the Control group than with the DSS group, consistent with a partial shift of the microbial profile toward that of the Control group following SQ treatment. Alpha-diversity analysis demonstrated that DSS-induced colitis reduced microbial richness and diversity, whereas SQ treatment partially restored these alterations (Figure 7B). Compared with the DSS group, SQ significantly increased the Shannon and Simpson diversity indices, while the Chao1 index also exhibited a recovery trend, indicating improved microbial richness and community diversity. Principal coordinate analysis (PCoA) further demonstrated a marked separation between the Control and DSS groups, indicating that DSS profoundly altered the overall microbial community structure (Figure 7C). In contrast, samples from the SQ-treated group clustered closer to those of the Control group along the principal coordinate axis, suggesting that SQ effectively remodeled DSS-induced microbial dysbiosis. Collectively, these findings indicate that SQ partially restored microbial richness, diversity, and overall community structure disrupted by DSS administration. Taxonomic composition analysis further demonstrated that SQ markedly remodeled the gut microbiota at both the phylum and genus levels (Figures 7D,E). At the phylum level, DSS administration markedly decreased the relative abundances of *Bacteroidota*, *Firmicutes_A*, *Patescibacteria*, *Cyanobacteria*, and *Firmicutes_B*, while increasing the abundances of *Proteobacteria*, *Verrucomicrobiota*, *Actinobacteriota*, and *Desulfobacterota_I*, indicating severe microbial dysbiosis. SQ treatment partially reversed several of these DSS-associated alterations, including recovery of *Bacteroidota*- and *Firmicutes*-related taxa and reductions in several DSS-enriched phyla. At the genus level, DSS exposure markedly reduced several commensal bacteria, including *Prevotella*, *UMGS1994*, *UBA7173*, *CAG-485*, *Duncaniella*, *Lactobacillus*, *Intestinimonas*, *UBA3282*, *Ligilactobacillus*, and *Paramuribaculum*, whereas the relative abundances of *Akkermansia*, *Escherichia*, *Bacteroides_H*, and *Parabacteroides_B* were significantly increased. SQ intervention substantially restored the abundance of several DSS-depleted commensal taxa, including *Prevotella*, *UMGS1994*, *UBA7173*, *Muribaculum*, *Lactobacillus*, *Intestinimonas*, and *Ligilactobacillus*, while reducing several DSS-enriched genera, including *Escherichia*, *Bacteroides_H*, *Parabacteroides_B*, and *Akkermansia*. Collectively, these findings indicate that SQ effectively remodeled DSS-disrupted gut microbial composition by enriching health-associated bacterial taxa while suppressing inflammation-related microorganisms. To further identify microbial biomarkers associated with SQ treatment, LEfSe analysis was performed using an LDA threshold of 4.0 (Figures 7F,G). The cladogram revealed distinct taxonomic enrichment patterns among the three groups. DSS administration was characterized by enrichment of several DSS-associated discriminative taxa, including *Proteobacteria*, *Verrucomicrobiota*, *Actinobacteriota*, and *Coriobacteriia*, whereas SQ treatment preferentially enriched *Firmicutes_A*, *Clostridia*, *Oscillospirales*, *Erysipelotrichales*, and *Muribaculaceae*, indicating a distinct microbial signature associated with SQ intervention. Consistently, LDA score analysis identified *Prevotella* and *Bacteroidota*-related taxa as discriminative taxa in the Control group, whereas *Akkermansia*, *Escherichia*, *Enterobacterales*, *Verrucomicrobiales*, and *Proteobacteria* were predominantly enriched in the DSS group. In contrast, the SQ group exhibited enrichment of *Muribaculaceae*, *Clostridia*, *Firmicutes_A*, *Erysipelotrichaceae*, and *Faecalibaculum*, indicating a distinct taxonomic signature associated with SQ treatment.

**FIGURE 7:**
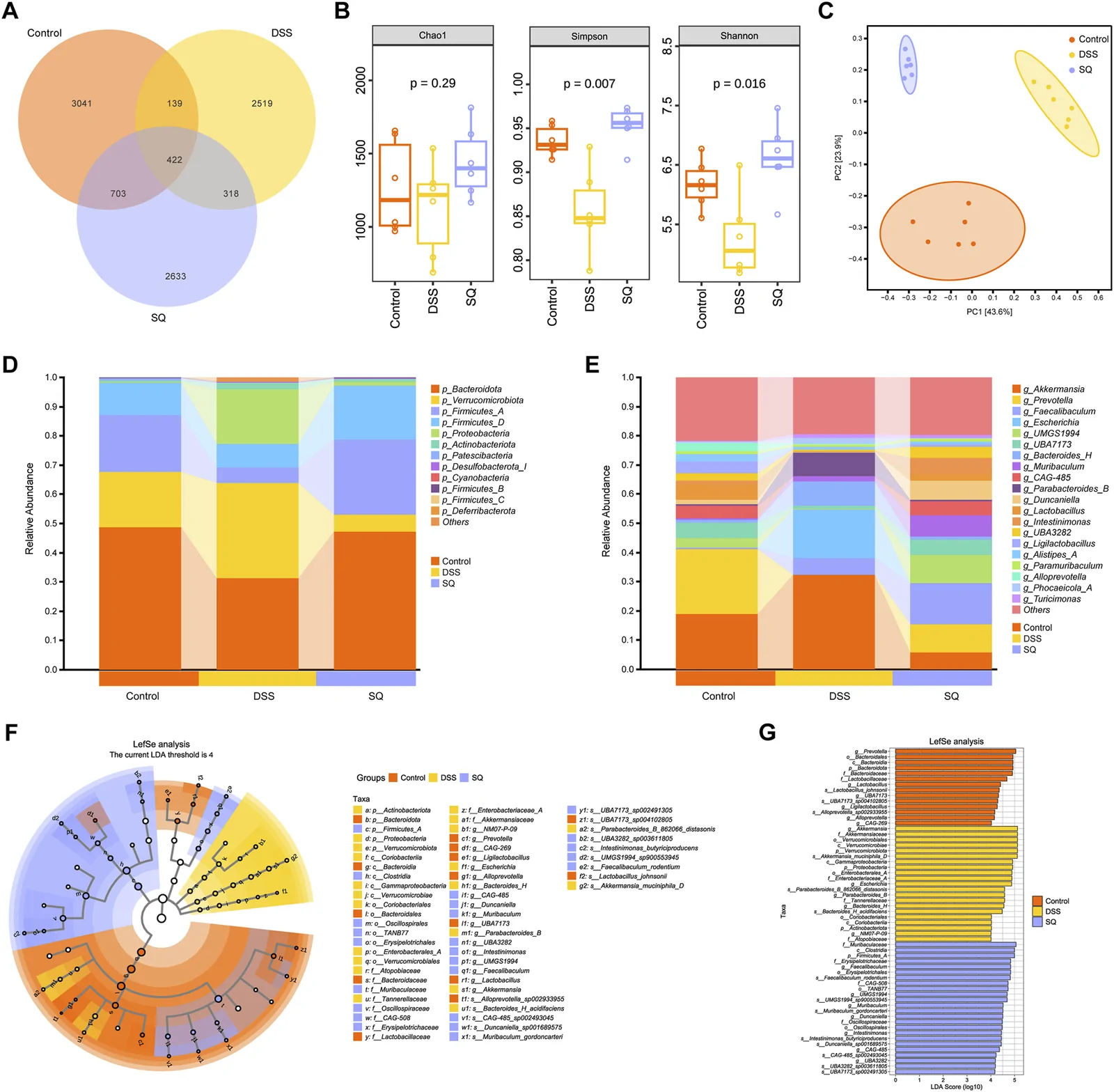
SQ remodels gut microbiota composition in DSS-induced colitis mice. **(A)** Venn diagram showing the shared and group-specific ASVs among the Control, DSS, and DSS + H-SQ (SQ) groups. **(B)** Alpha-diversity analysis of gut microbiota based on the Chao1, Shannon, and Simpson indices. **(C)** Principal coordinate analysis (PCoA) of β-diversity showing the overall differences in microbial community structure among the three groups. **(D)** Relative abundance of gut microbiota at the phylum level. **(E)** Relative abundance of gut microbiota at the genus level. **(F)** LEfSe cladogram illustrating differentially enriched microbial taxa among the three groups. **(G)** Linear discriminant analysis (LDA) score distribution of significantly enriched microbial taxa (LDA >4.0). Data are presented as the mean ± SD (n = 6). ^*^ *P* < 0.05.

### 3.9 Microbiota depletion markedly attenuates the protective effects of SQ against DSS-induced colitis

To determine whether the anti-colitis effect of SQ is dependent on the gut microbiota, an antibiotic (ABX)-induced microbiota depletion model was established (Figure 8A). Quantification of fecal DNA revealed a marked reduction in microbial biomass following ABX treatment compared with non-ABX mice, confirming effective depletion of gut microbiota (Figure 8B). Under microbiota-depleted conditions, DSS administration induced typical colitis manifestations, including colon shortening, body weight loss, and elevated disease activity index (DAI) scores (Figures 8C–F). Notably, high-dose SQ treatment did not significantly improve these parameters, as no significant differences were observed between the DSS and DSS + H-SQ groups in colon length, body weight change, or DAI scores. Consistently, histopathological analysis showed that DSS caused severe colonic injury characterized by epithelial disruption, inflammatory cell infiltration, and mucosal damage, which were not significantly ameliorated by SQ treatment in ABX-treated mice (Figures 8G,H). In parallel, DSS-induced increases in pro-inflammatory cytokines (IL-1β, IL-6, and TNF-α) and the reduction in anti-inflammatory IL-10 levels were not significantly reversed by SQ administration (Figures 8I–L). Collectively, these results indicate that antibiotic-mediated microbiota depletion markedly attenuated the protective effects of SQ against DSS-induced colitis, supporting an important functional contribution of the gut microbiota to SQ-mediated protection.

**FIGURE 8:**
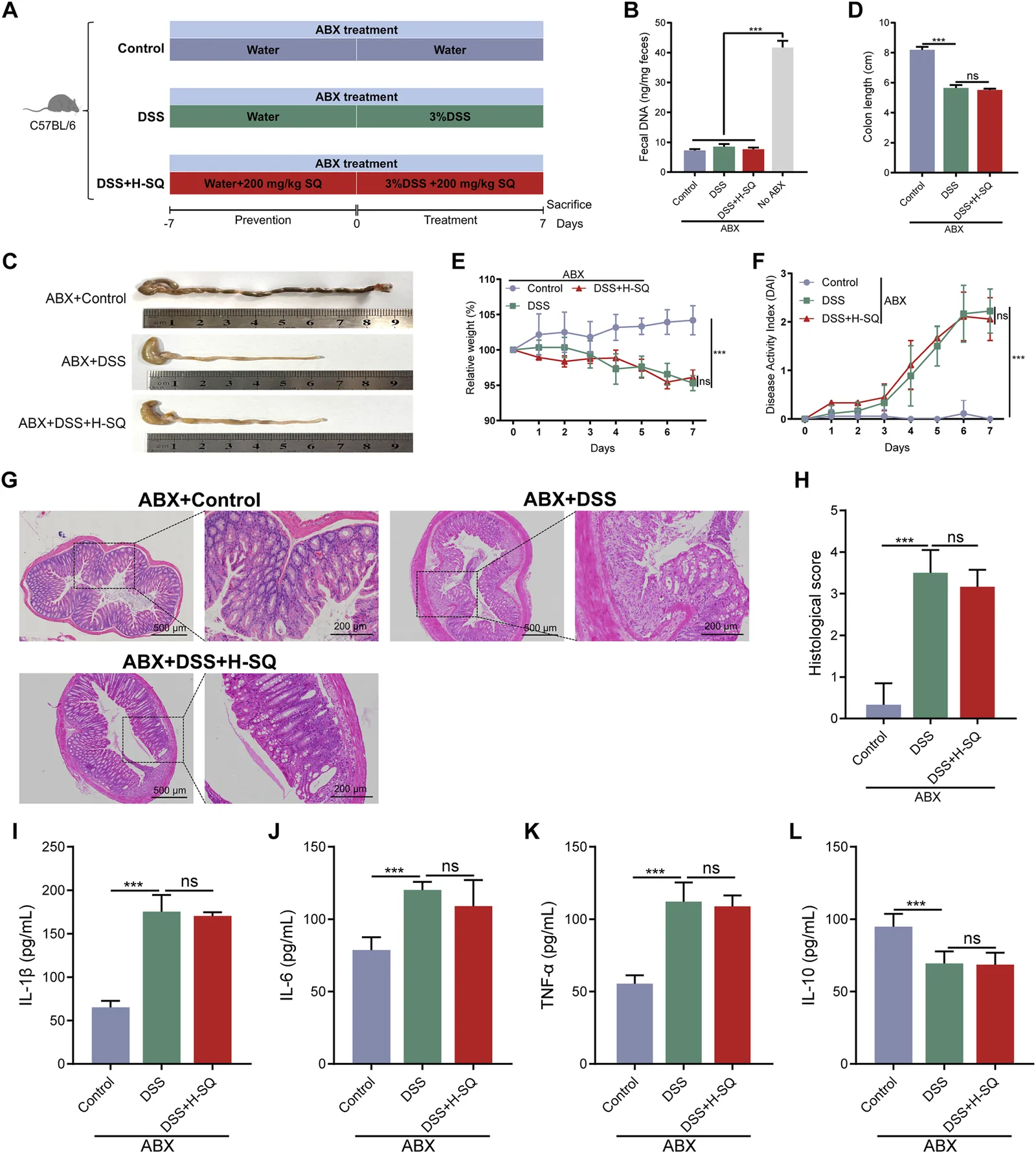
Gut microbiota depletion markedly attenuates the protective effects of SQ in DSS-induced colitis. **(A)** Experimental design of antibiotic (ABX)-induced gut microbiota depletion and subsequent DSS and SQ treatment. **(B)** Quantification of fecal DNA content (ng/mg feces) to assess the efficiency of microbiota depletion. **(C)** Representative images of colons from each group. **(D)** Colon length of mice in each group. **(E)** Changes in body weight relative to initial weight (day 0). **(F)** Disease activity index (DAI) scores. **(G)** Representative H&E staining images of colon tissues. Scale bars = 500 μm (left panels) and 200 μm (right panels). **(H)** Histopathological scores of colon tissues. **(I–L)** Levels of inflammatory cytokines IL-1β **(I)**, IL-6 **(J)**, TNF-α **(K)**, and IL-10 **(L)** in colon tissues determined by ELISA. Data are presented as mean ± SD (n = 6). ^***^ *P* < 0.001. ns, not significant.

### 3.10 FMT transfers SQ-associated protection against DSS-induced colitis

To further determine whether the protective effects of SQ against colitis are mediated by the gut microbiota, a fecal microbiota transplantation (FMT) experiment was performed using donor microbiota derived from Control, DSS, and DSS + H-SQ-treated mice (Figure 9A). Prior to transplantation, antibiotic treatment effectively depleted endogenous microbiota in recipient mice, as evidenced by a marked reduction in fecal DNA content (Figure 9B), thereby establishing a microbiota-depleted recipient background. Following DSS induction, recipient mice that received microbiota from DSS donors (FMT-DSS group) exhibited typical colitis phenotypes, including colon shortening, progressive body weight loss, and elevated DAI scores (Figures 9C–F). In contrast, mice transplanted with microbiota from SQ-treated donors (FMT-DSS + H-SQ group) displayed significantly alleviated disease severity, as reflected by increased colon length, improved body weight maintenance, and reduced DAI scores. Histopathological examination further supported these findings, showing that FMT from SQ-treated donors markedly attenuated DSS-induced mucosal injury, inflammatory cell infiltration, and structural disruption compared with the FMT-DSS group (Figure 9G,H). Consistently, the levels of pro-inflammatory cytokines (IL-1β, IL-6, and TNF-α) were significantly reduced, whereas the anti-inflammatory cytokine IL-10 was elevated in the FMT-DSS + H-SQ group (Figures 9I–L). Collectively, these findings demonstrate that FMT from SQ-treated donors transferred a protective phenotype against DSS-induced colitis, supporting a functional contribution of SQ-associated gut microbiota remodeling to intestinal protection. Thus, a functionally important component of SQ-mediated protection is transferable through the gut microbiota.

**FIGURE 9:**
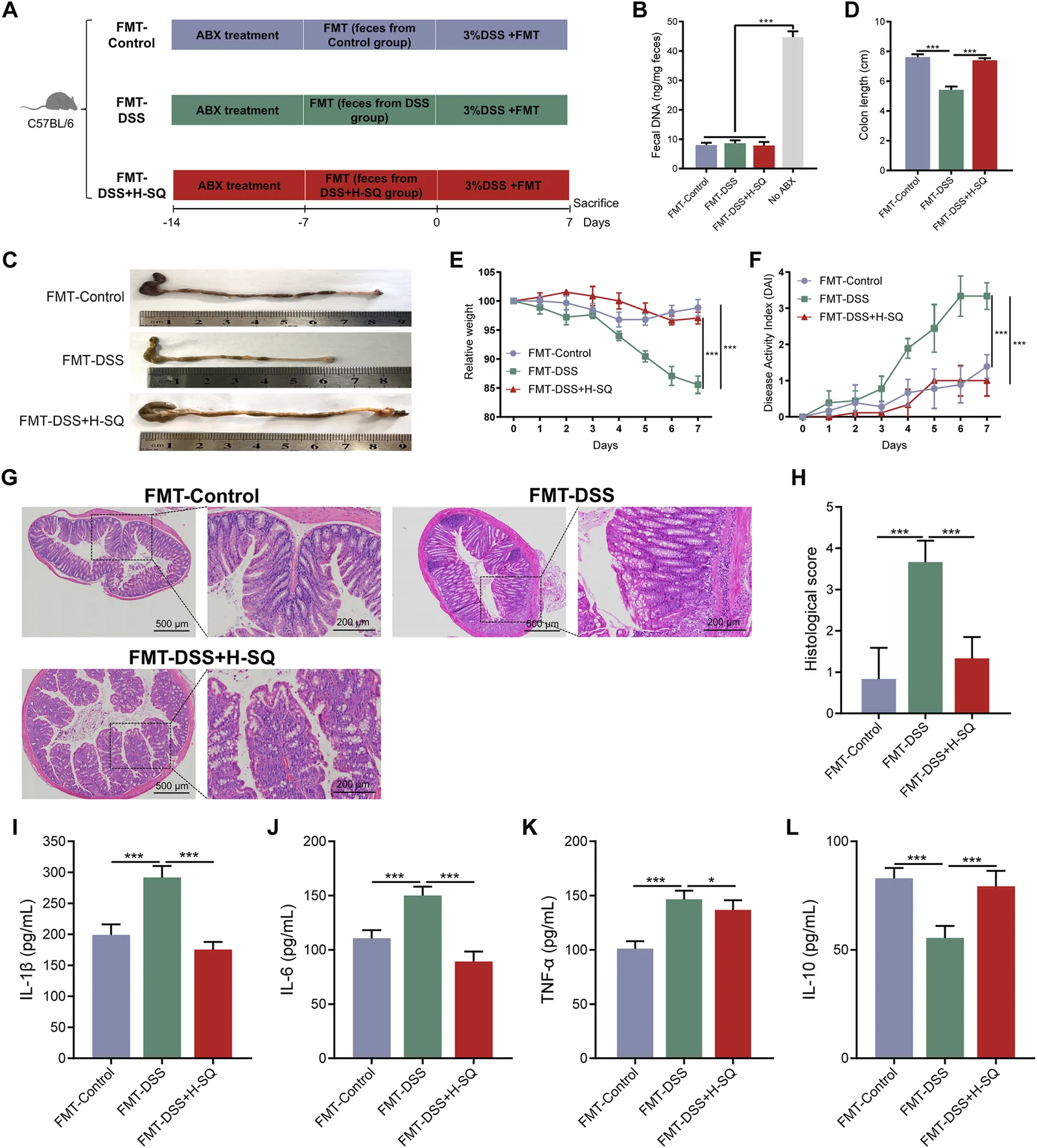
FMT transfers SQ-associated protection against DSS-induced colitis. **(A)** Experimental design of fecal microbiota transplantation (FMT) using donor microbiota derived from Control, DSS, and DSS + H-SQ-treated mice. **(B)** Quantification of fecal DNA content (ng/mg feces) to verify microbiota depletion in recipient mice following antibiotic treatment. **(C)** Representative images of colons from each group. **(D)** Colon length of mice in each group. **(E)** Changes in body weight relative to initial weight (day 0). **(F)** Disease activity index (DAI) scores. **(G)** Representative H&E staining images of colon tissues. Scale bars = 500 μm (left panels) and 200 μm (right panels). **(H)** Histopathological scores of colon tissues. **(I–L)** Levels of inflammatory cytokines IL-1β **(I)**, IL-6 **(J)**, TNF-α **(K)**, and IL-10 **(L)** in colon tissues determined by ELISA. Data are presented as mean ± SD (n = 6). ^*^ *P* < 0.05. ^***^ *P* < 0.001.

## 4 Discussion

Ulcerative colitis (UC) is a chronic relapsing inflammatory bowel disease characterized by a complex interplay of excessive inflammation, intestinal barrier dysfunction, and oxidative stress (Jia et al., 2026). Persistent activation of pro-inflammatory pathways, particularly nuclear factor-κB (NF-κB), drives the overproduction of cytokines such as IL-1β, IL-6, and TNF-α, thereby exacerbating mucosal injury. Meanwhile, disruption of epithelial tight-junction integrity compromises barrier function, facilitating luminal antigen translocation and amplifying inflammatory responses. In parallel, oxidative stress further contributes to epithelial damage and disease progression, with the nuclear factor erythroid 2-related factor 2 (Nrf2) signaling pathway serving as a central regulator of endogenous antioxidant defense and redox homeostasis (Nguyen et al., 2009). Given this multifactorial pathogenesis, therapeutic strategies capable of simultaneously modulating inflammation, restoring barrier function, and rebalancing redox homeostasis are of particular interest.

Natural bioactive compounds have emerged as promising candidates for UC management due to their multitarget properties. Squalene (SQ), a natural triterpenoid widely distributed in plants and animals, possesses antioxidant, anti-inflammatory, and cytoprotective activities (Micera et al., 2020; Luke et al., 2024). Previous work has already demonstrated that dietary SQ can attenuate DSS-induced acute colitis, with its protective effects primarily associated with suppression of p38 MAPK/NF-κB-mediated inflammatory signaling (Sanchez-Fidalgo et al., 2015). However, whether SQ-mediated protection depends on a defined host molecular pathway and whether the gut microbiota contributes functionally, rather than merely associatively, to this protection remain unresolved. The 50 and 200 mg/kg doses were selected to span a pharmacologically active range reported in previous oral rodent studies (Sakul et al., 2019; Kim et al., 2026), although these experimental exposures should not be directly extrapolated to habitual human SQ intake. Although previous studies suggest that orally administered SQ is generally well tolerated, dedicated long-term dose-ranging safety studies at pharmacologically active exposures remain limited (Smith, 2000; Cho et al., 2009); therefore, the present findings should not be interpreted as establishing the chronic safety of SQ.

In the present study, we systematically evaluated the protective effects of SQ in DSS-induced colitis through a combination of *in vivo* and *in vitro* approaches, together with mechanistic investigations involving Nrf2-deficient mice, antibiotic-mediated microbiota depletion, and fecal microbiota transplantation (FMT). Our results demonstrate that SQ markedly alleviates DSS-induced colitis by improving disease manifestations, attenuating histopathological damage, suppressing inflammatory responses, restoring intestinal barrier integrity, and enhancing antioxidant capacity. Importantly, an independent positive-control experiment showed that H-SQ produced marked improvements across major clinical, histopathological, and inflammatory endpoints when evaluated alongside 5-ASA. Although the present study was not designed as a formal equivalence or non-inferiority comparison, this pharmacological benchmark provides additional context for the magnitude of SQ-mediated protection and strengthens its preclinical translational relevance. Mechanistically, the marked attenuation of SQ-mediated protection in Nrf2-deficient mice, together with the effects of pharmacological Nrf2 inhibition *in vitro*, supports Nrf2 as the principal host molecular axis underlying the protective actions of SQ. In parallel, antibiotic-mediated microbiota depletion markedly weakened SQ-mediated protection, whereas FMT from SQ-treated donors transferred protection to recipient mice, supporting an important functional contribution of gut microbiota remodeling. Thus, the current evidence supports a mechanistic framework in which Nrf2 represents the principal host molecular mediator, while microbiota remodeling constitutes a complementary functional component of SQ-mediated intestinal protection. However, the present study does not establish a direct upstream-downstream relationship between these two components. Accordingly, the present study extends previous efficacy-focused observations by providing functional mechanistic evidence that supports Nrf2 as a principal host mediator and gut microbiota remodeling as an important contributor to SQ-mediated intestinal protection.

Aberrant activation of the nuclear factor-κB (NF-κB) signaling pathway is a central driver of mucosal inflammation in UC, orchestrating the transcription of a wide range of pro-inflammatory mediators, including cytokines and enzymes such as cyclooxygenase-2 (COX-2) (Jia et al., 2026). Under inflammatory stimulation, activation of canonical NF-κB signaling promotes IκBα phosphorylation and degradation, allowing NF-κB complexes containing p65 to translocate to the nucleus and drive pro-inflammatory gene transcription (Mukherjee et al., 2024). In the present study, DSS exposure markedly activated the NF-κB pathway, as evidenced by increased phosphorylation of IκBα and p65, accompanied by elevated COX-2 expression and elevated levels of pro-inflammatory cytokines, including IL-1β, IL-6, and TNF-α. Notably, SQ treatment significantly suppressed NF-κB activation, as reflected by reduced phosphorylation of IκBα and p65, and concomitantly downregulated COX-2 expression. This inhibitory effect was further supported by a pronounced decrease in pro-inflammatory cytokines and a partial restoration of the anti-inflammatory cytokine IL-10. These findings indicate that SQ attenuates DSS-induced intestinal inflammation at least in part through suppression of canonical NF-κB signaling, thereby limiting downstream inflammatory mediator production. Consistent with previous reports on natural terpenoids and lipophilic bioactive compounds (Jiang et al., 2023; Zhang T. et al., 2024), which frequently exert anti-inflammatory effects via modulation of NF-κB signaling, our findings support an anti-inflammatory role for SQ in experimental colitis.

Intestinal barrier dysfunction is increasingly recognized as a key pathogenic feature of ulcerative colitis, serving as a critical interface through which luminal microbial stimuli perpetuate mucosal inflammation (Peterson and Artis, 2014). This barrier relies on the coordinated function of intercellular tight junctions and the mucus layer, which together restrict antigen translocation and maintain epithelial homeostasis. Disruption of tight junction proteins, such as ZO-1 and occludin, compromises paracellular sealing, while depletion or dysfunction of goblet cells impairs MUC2-dependent mucus coverage, collectively facilitating microbial encroachment and sustained immune activation (Johansson, 2014; Zhang H. et al., 2024). Within this framework, SQ not only restored the expression of tight junction proteins but also functionally reinforced epithelial barrier integrity, as evidenced by increased transepithelial electrical resistance and reduced FITC-dextran permeability in Caco-2 monolayers. Together with the recovery of goblet cell-associated mucus features *in vivo*, these findings suggest coordinated reinforcement of both the epithelial and mucus barriers. Rather than reflecting isolated molecular or structural alterations, these findings indicate that SQ preserves epithelial barrier function through coordinated maintenance of tight junction integrity and mucus homeostasis. Such integrated barrier protection is mechanistically relevant because inflammatory cytokines and oxidative stress are known to disrupt junctional organization, increase epithelial permeability, and impair mucin secretion, thereby establishing a self-perpetuating cycle of barrier dysfunction and mucosal inflammation (Huo et al., 2022). Preservation of this epithelial–mucus barrier system may therefore limit luminal antigen access and contribute to the re-establishment of mucosal homeostasis at the host–microbe interface.

Oxidative stress is increasingly recognized as a central driver in the initiation and perpetuation of colitis, as excessive reactive oxygen species (ROS) disrupt epithelial integrity, amplify inflammatory signaling, and sustain mucosal injury (Bhattacharyya et al., 2014). In this context, the Nrf2 signaling pathway functions as a master regulator of cellular redox homeostasis by coordinating the transcription of a broad set of antioxidant and cytoprotective genes, including HO-1 and NQO1 (Nguyen et al., 2009). Dysfunction of this pathway has been closely linked to uncontrolled oxidative damage and disease progression in UC (Jiang et al., 2024; Zhang T. et al., 2024). Within this framework, the redox imbalance observed under colitic conditions was accompanied by suppression of endogenous antioxidant systems and accumulation of lipid peroxidation products, whereas activation of the Nrf2 axis was associated with restoration of antioxidant enzyme activities (e.g., SOD, CAT, and GSH-Px) and reduction of oxidative injury markers such as MDA. Rather than representing isolated biochemical changes, this coordinated shift reflects a re-establishment of intestinal redox homeostasis, which is particularly relevant given the tight interplay between oxidative stress, inflammation, and epithelial barrier disruption. Notably, complementary *in vitro* evidence further supports the functional involvement of Nrf2, as pharmacological inhibition of Nrf2 largely attenuated the antioxidant and cytoprotective effects of SQ, while SQ treatment enhanced the thermal stability of Nrf2 in CETSA assays, consistent with potential target engagement. Importantly, the incorporation of Nrf2-deficient models provides critical mechanistic insight beyond descriptive association. Under identical DSS challenge conditions, the marked attenuation of SQ-mediated protection in Nrf2-deficient mice indicates that the observed redox reprogramming and downstream tissue protection are largely dependent on an intact Nrf2 signaling cascade, rather than reflecting nonspecific antioxidant effects alone. Although Nrf2 deficiency itself has been reported to influence basal redox homeostasis and intestinal physiology, the comparative responses observed under the same experimental conditions further support Nrf2 as a principal mediator of SQ-mediated protection. Collectively, the genetic and pharmacological loss-of-function evidence supports Nrf2 as the principal host molecular axis underlying SQ-mediated protection, integrating antioxidant defense with inflammatory resolution and epithelial barrier preservation during colitis.

Gut microbial dysbiosis is widely recognized as a hallmark of UC and contributes to disease progression through reciprocal interactions with intestinal inflammation, epithelial barrier dysfunction, and oxidative stress (Sartor, 2008; Bethlehem et al., 2024). Consistent with previous studies, DSS markedly reduced microbial diversity and altered community structure, accompanied by expansion of inflammation-associated taxa such as *Proteobacteria* and *Escherichia* and depletion of multiple commensal taxa. SQ partially reversed these alterations, partially restoring microbial diversity and shifting the microbial profile toward that of the control group. Several taxa affected by SQ have previously been associated with microbial metabolic functions, including SCFA metabolism and maintenance of mucosal homeostasis (Markowiak-Kopec and Slizewska, 2020); however, SCFAs and other microbiota-derived metabolites were not directly measured in the present study. Therefore, these taxonomic changes should be interpreted as evidence of microbial ecological remodeling rather than direct evidence of altered microbial metabolic output. The observed microbiota remodeling may also partly reflect an improved intestinal environment resulting from reduced oxidative stress and inflammation.

Beyond the compositional changes revealed by 16S rRNA sequencing, the ABX depletion and FMT experiments provide functional evidence that the gut microbiota contributes to SQ-mediated protection against colitis. Antibiotic-mediated microbiota depletion markedly weakened the ability of SQ to improve disease severity, histological injury, and inflammatory responses. Conversely, transplantation of microbiota from SQ-treated donors transferred protection against DSS-induced colitis to recipient mice. Together, these findings support a functional contribution of SQ-associated microbiota remodeling rather than a purely secondary association with reduced intestinal inflammation. However, the microbiota-derived signals responsible for this transferable effect remain unresolved. SCFAs are established microbiota-derived mediators of epithelial and immune homeostasis in other experimental settings, but their involvement in the effects observed here remains hypothetical because neither SCFAs nor other microbial metabolites were directly quantified. Accordingly, the present data support gut microbiota remodeling as an important complementary functional component of SQ-mediated protection, while Nrf2 appears to constitute the principal host molecular regulatory axis. The metabolic intermediates and directionality of the interaction between these two components remain to be established.

From a translational perspective, several pathological features ameliorated by SQ in the present study—including mucosal epithelial injury, crypt disruption, inflammatory cell infiltration, impaired epithelial and mucus barrier integrity, inflammatory cytokine dysregulation, oxidative stress, and microbial dysbiosis—overlap with clinically relevant features of human UC. Nevertheless, acute DSS colitis is initiated by chemical epithelial injury and does not fully reproduce the chronic relapsing course or adaptive immune complexity of human UC; therefore, the present efficacy findings should not be directly extrapolated to patients (Yang and Merlin, 2024). Given the preventive and continuous intervention schedule used here and the protective efficacy observed alongside 5-ASA, future clinical evaluation, if supported by pharmacokinetic and safety studies, may be more appropriately considered initially in adults with mild-to-moderate UC as an adjunct to standard therapy or potentially in maintenance or relapse-prevention settings, rather than in acute severe UC. Oral administration represents a practical route for further development; however, because SQ is highly lipophilic and poorly water-soluble, lipid-based formulations, emulsions, nanoemulsions, or other encapsulation approaches may warrant investigation to improve its dispersion, stability, and gastrointestinal delivery (McClements, 2013; Lekshmi et al., 2019; Kumar et al., 2026). These translational possibilities remain hypothetical and require validation in chronic or relapsing models, pharmacokinetic and long-term safety studies, and ultimately well-designed clinical trials.

Several limitations of the present study should be acknowledged. First, the protective effects of SQ were evaluated primarily in an acute DSS-induced colitis model with a relatively short intervention period. The present design was not intended as a formal toxicity study and did not include dedicated healthy SQ-treated cohorts for long-term dose-ranging safety assessment. Therefore, whether the protective effects are sustained in chronic or relapsing colitis, and whether prolonged exposure to pharmacologically active doses of SQ affects hematological, biochemical, or major organ safety parameters, remain to be established. Second, although 16S rRNA sequencing, antibiotic-mediated microbiota depletion, and FMT collectively support an important contribution of the gut microbiota to SQ-mediated protection, microbiota-derived metabolites, including short-chain fatty acids, were not directly quantified, nor was metabolomic profiling performed. Accordingly, the proposed links between specific microbial alterations and host redox or inflammatory signaling should be regarded as biologically plausible hypotheses rather than directly demonstrated metabolic mechanisms. Third, the present experimental design does not establish the temporal or hierarchical relationship between Nrf2 activation and gut microbiota remodeling; therefore, potential bidirectional interactions between these two components warrant further investigation. In addition, Caco-2 cells represent a simplified epithelial model and cannot fully reproduce the multicellular complexity of the intestinal mucosa. Finally, CETSA, molecular docking, and molecular dynamics simulations provide complementary evidence supporting a potential interaction between SQ and Nrf2; however, definitive characterization of direct binding and binding kinetics will require orthogonal biophysical approaches. Future studies incorporating chronic or relapsing colitis models, pharmacokinetic evaluation, targeted quantification of microbiota-derived metabolites together with metabolomic profiling, optimized oral formulation development, primary intestinal epithelial or organoid models, and longer-term dose-ranging safety assessments will help further define the mechanistic and translational relevance of SQ.

## 5 Conclusion

Collectively, the present study demonstrates that SQ protects against DSS-induced colitis by attenuating intestinal inflammation and oxidative stress, preserving epithelial barrier integrity, and remodeling the gut microbiota. Mechanistically, SQ-mediated protection was strongly dependent on Nrf2 signaling, supporting Nrf2 as the principal host molecular axis underlying the protective effects of SQ, whereas microbiota depletion and FMT experiments supported an important complementary functional contribution of gut microbiota remodeling. These findings support a mechanistic framework in which Nrf2-dependent host defense represents the central molecular component, with a functionally important microbiota-associated contribution, and warrant further investigation of SQ as a potential intervention for UC and related intestinal inflammatory disorders.

## Data Availability

The raw data supporting the conclusions of this article will be made available by the authors, without undue reservation.
