# Supplementary material for: Squalene alleviates DSS-induced colitis by restoring Nrf2-dependent redox balance and gut microbiota homeostasis
Source: Front Pharmacol. 2026 Sep 18;17:1938047. doi: 10.3389/fphar.2026.1938047 (PMC13630718; doi:10.3389/fphar.2026.1938047)
Supplement: Supplementary file 1 [file Table1.docx]

***Supplementary Material***

**Title: Squalene protects against DSS-induced colitis by orchestrating Nrf2-mediated antioxidant defense and gut microbiota homeostasis**

Peng Jiang^1,4^, Zhao Ling^1,2^, Shuo Han^1,2^, Yake Wang^1,2*^, Jing Wang^1,2,3*^

1. College of Animal Science and Technology, Tarim University, Alaer, 843300, China

2. Engineering Laboratory of Tarim Animal Diseases Diagnosis and Control, Xinjiang Production & Construction Corps, Alaer, 843300, China

3. Hangzhou Xiaoshan Donghai Aquaculture Co.Ltd, Hangzhou, Zhejiang Province 311200, China

4. State Key Laboratory for Diagnosis and Treatment of Severe Zoonotic Infectious Diseases, Institute of Zoonosis, and College of Veterinary Medicine, Jilin University,
Changchun, 130062, China

^*^Corresponding author

*E-mail* address: [mswyk1314@163.com](mailto:mswyk1314@163.com); [wangjing2050@163.com](mailto:wangjing2050@163.com)

**Supplementary Table S1. Primary antibodies used for Western blotting**

| **Target** | **Manufacturer** | **Cat. No.** | **Dilution** |
| --- | --- | --- | --- |
| GAPDH | Abcam | ab8245 | 1:5,000 |
| p65 | Abcam | ab16502 | 1:2,000 |
| p-p65 | Abcam | ab76302 | 1:1,000 |
| IκB | Abcam | ab32518 | 1:2,000 |
| p-IκB | Abcam | ab133462 | 1:4,000 |
| Nrf2 | Abcam | ab313825 | 1:1,000 |
| COX-2 | Proteintech | 12375-1-AP | 1:1,000 |
| ZO-1 | Proteintech | 21773-1-AP | 1:5,000 |
| Occludin | Proteintech | 27260-1-AP | 1:5,000 |
| HO-1 | Proteintech | 10701-1-AP | 1:5,000 |
| NQO1 | Proteintech | 86693-2-RR | 1:5,000 |

**Supplementary Table S2. DAI scoring criteria**

| **Score** | **Weight loss (%)** | **Stool consistency** | **Occult blood test** |
| --- | --- | --- | --- |
| 0 | None | Normal | No bleeding |
| 1 | 1-5 | Soft but still formed | Positive hemoccult |
| 2 | 6-10 | Soft | Visible blood traces in stool |
| 3 | 11-15 | Very soft; wet | Visible anal blood traces |
| 4 | ＞15 | Watery diarrhea | Gross bleeding |

**Supplementary Table S3. Histopathological scoring criteria**

| **Score** | **Inflammation** | **Extent** | **Crypt damage** |
| --- | --- | --- | --- |
| 0 | None | None | None |
| 1 | Slight | Mucosa | Basal 1/3 damaged |
| 2 | Moderate | Mucosa and submucosa | Basal 2/3 damaged |
| 3 | Severe | Transmural | Complete crypt loss with intact surface epithelium |
| 4 |  |  | Entire crypt and surface epithelium loss |

**Supplementary Table S4. Primer sequences used for qPCR**

| **Primers** | **Forward (5′ to 3′)** | **Reverse (5′ to 3′)** |
| --- | --- | --- |
| IL-1β | TGCCACCTTTTGACAGTGATG | AAGGTCCACGGGAAAGACAC |
| IL-6 | ACAGCCACTCACCTCTTCAG | CCATCTTTTTCAGCCATCTTT |
| TNF-α | GCTGCACTTTGGAGTGATCG | ATGAGGTACAGGCCCTCTGA |
| GAPDH | TCAAGAAGGTGGTGAAGCAGG | TCAAGGTGGAGGAGTGGGT |

**Supplementary Fig. S1. Overview of the animal experimental designs used in this study.** Five independent animal experiments were conducted to evaluate the protective effects and underlying mechanisms of squalene (SQ) in DSS-induced colitis. (i) SQ dose-response experiment in C57BL/6 mice, including Control, DSS, DSS+L-SQ (50 mg/kg), and DSS+H-SQ (200 mg/kg) groups. SQ was administered by oral gavage for 7 days before DSS exposure and continued during the subsequent 7-day period of 3% DSS administration. (ii) Positive-control validation experiment comparing H-SQ (200 mg/kg) with 5-aminosalicylic acid (5-ASA, 100 mg/kg) under the same DSS challenge schedule. (iii) Nrf2-dependency experiment using WT and Nrf2-KO mice, in which each genotype was assigned to DSS and DSS+H-SQ groups. (iv) Antibiotic-mediated microbiota depletion experiment in which mice received a broad-spectrum antibiotic cocktail throughout the experimental period, with or without H-SQ treatment and subsequent DSS challenge. (v) Fecal microbiota transplantation (FMT) experiment. Recipient mice were first pretreated with antibiotics, followed by transplantation of fecal microbiota derived from Control, DSS, or DSS+H-SQ donor mice; DSS was administered during the final 7 days while FMT was continued. All mice were euthanized at the indicated experimental endpoints. L-SQ, low-dose squalene; H-SQ, high-dose squalene; DSS, dextran sulfate sodium; 5-ASA, 5-aminosalicylic acid; ABX, antibiotic cocktail; FMT, fecal microbiota transplantation; WT, wild type; Nrf2-KO, Nrf2 knockout.

**Supplementary Fig. S2. Comparison of the protective effects of H-SQ and 5-ASA in DSS-induced colitis.** (A) Schematic illustration of the experimental design. Mice were assigned to Control, DSS, DSS+H-SQ (200 mg/kg), and DSS+5-ASA (100 mg/kg) groups. H-SQ or 5-ASA was administered for 7 days before DSS exposure and continued during the subsequent 7-day period of 3% DSS administration. (B) Changes in relative body weight during the experimental period. (C) Disease activity index (DAI) scores. (D) Representative images of colons from each group. (E) Quantitative analysis of colon length. (F) Representative hematoxylin and eosin (H&E) staining of colonic tissues. The right panels show enlarged views of the boxed regions in the corresponding left panels. Scale bars = 500 μm (left panels) and 200 μm (right panels). (G) Histopathological scores of colonic injury. (H–K) Colonic levels of IL-1β (H), IL-6 (I), TNF-α (J), and IL-10 (K), determined by ELISA. Data are presented as mean ± SD (n = 6). ^###^*P* < 0.001 vs. Control group; ^*^*P* < 0.05, ^**^*P* < 0.01, ^***^*P* < 0.001 vs. DSS group. Horizontal brackets indicate direct comparisons between the DSS+H-SQ and DSS+5-ASA groups; ns, not significant.

**Supplementary Fig. S3. SQ preserves epithelial barrier function in LPS-stimulated Caco-2 monolayers.** (A) Schematic illustration of the experimental design for transepithelial electrical resistance (TEER) and FITC-dextran permeability assays using differentiated Caco-2 monolayers cultured on Transwell inserts. (B) Relative TEER values (% of control) in Caco-2 monolayers under the indicated treatments. (C) Relative FITC-dextran permeability (% of control) in Caco-2 monolayers under the indicated treatments. Data are presented as mean ± SD (n = 3). ^###^*P* < 0.001 vs. Control group. ^*^*P* < 0.05, ^***^*P* < 0.001 vs. LPS group.

**Supplementary Fig. S4. SQ activates Nrf2 signaling and exerts antioxidant effects in an Nrf2-dependent manner in Caco-2 cells.** (A) Representative Western blot images of Nrf2, HO-1, and NQO1 in Caco-2 cells under the indicated treatments. (B-D) Quantitative analysis of Nrf2, HO-1, and NQO1 protein levels. (E) CETSA analysis of Nrf2 protein stability in Caco-2 cells treated with SQ or DMSO. (F) Relative Nrf2 band intensity (%) at different temperatures. (G-H) SOD activity and MDA levels in Caco-2 cells. (I) Cell viability assessed by CCK-8 assay. Data are presented as mean ± SD (n = 3). ^**^*P* < 0.01. ^***^*P* < 0.001.

**Supplementary Fig. S5. Structural and energetic characterization of the predicted interaction between SQ and Nrf2.** (A) Representative overall structure of the Nrf2–SQ complex obtained from the 100-ns molecular dynamics (MD) trajectory at 85.25 ns. (B) Enlarged view of the SQ-associated binding region in the representative MD conformation. (C) Two-dimensional interaction map showing residues surrounding SQ within the remodeled binding region. (D) Residue-wise MM-PBSA energy decomposition showing major favorable and unfavorable energetic contributions to the Nrf2–SQ interaction. (E) MM-PBSA energy components of the Nrf2–SQ complex calculated from 101 frames extracted from the final 10 ns of the MD trajectory. ΔVDWAALS, van der Waals energy; ΔEEL, electrostatic energy; ΔEPB, polar solvation energy; ΔENPOLAR, nonpolar solvation energy; ΔGGAS, gas-phase molecular mechanics energy; ΔGSOLV, solvation energy; ΔTOTAL, estimated total binding energy.

**Supplementary Fig. S6. Dynamic behavior of the Nrf2–SQ complex during the 100-ns molecular dynamics simulation.** (A) Backbone root-mean-square deviation (RMSD) of Nrf2 during the 100-ns MD simulation. (B) RMSD of SQ calculated after alignment of the trajectory to the Nrf2 backbone. (C) Center-of-mass (COM) distance between SQ and the key binding region during the simulation. (D) Minimum heavy-atom distance between SQ and residues defining the key binding region, including HIS446, ARG449, LEU450, HIS453, GLU471, and ILE474. (E) Radius of gyration (Rg) of Nrf2. (F) Solvent-accessible surface area (SASA) of Nrf2. (G) Cα root-mean-square fluctuation (RMSF) of individual Nrf2 residues during the simulation. (H) Free-energy landscape of the Nrf2–SQ system constructed using backbone RMSD and Rg as reaction coordinates. Where indicated, mean values were calculated over the 50–100 ns interval.
